# A computational framework to assess genome-wide distribution of polymorphic human endogenous retrovirus-K In human populations

**DOI:** 10.1371/journal.pcbi.1006564

**Published:** 2019-03-28

**Authors:** Weiling Li, Lin Lin, Raunaq Malhotra, Lei Yang, Raj Acharya, Mary Poss

**Affiliations:** 1 The School of Electrical Engineering and Computer Science, The Pennsylvania State University, University Park, PA, United States of America; 2 Department of Statistics, The Pennsylvania State University, University Park, PA, United States of America; 3 Department of Biology, The Pennsylvania State University, University Park, PA, United States of America; 4 School of Informatics, Computing and Engineering, Indiana University, Bloomington, IN, United States of America; 5 Department of Veterinary and Biomedical Sciences, The Pennsylvania State University, University Park, PA, United States of America; University of Texas at Austin, UNITED STATES

## Abstract

Human Endogenous Retrovirus type K (HERV-K) is the only HERV known to be insertionally polymorphic; not all individuals have a retrovirus at a specific genomic location. It is possible that HERV-Ks contribute to human disease because people differ in both number and genomic location of these retroviruses. Indeed viral transcripts, proteins, and antibody against HERV-K are detected in cancers, auto-immune, and neurodegenerative diseases. However, attempts to link a polymorphic HERV-K with any disease have been frustrated in part because population prevalence of HERV-K provirus at each polymorphic site is lacking and it is challenging to identify closely related elements such as HERV-K from short read sequence data. We present an integrated and computationally robust approach that uses whole genome short read data to determine the occupation status at all sites reported to contain a HERV-K provirus. Our method estimates the proportion of fixed length genomic sequence (*k-mers*) from whole genome sequence data matching a reference set of *k-mers* unique to each HERV-K locus and applies mixture model-based clustering of these values to account for low depth sequence data. Our analysis of 1000 Genomes Project Data (KGP) reveals numerous differences among the five KGP super-populations in the prevalence of individual and co-occurring HERV-K proviruses; we provide a visualization tool to easily depict the proportion of the KGP populations with any combination of polymorphic HERV-K provirus. Further, because HERV-K is insertionally polymorphic, the genome burden of known polymorphic HERV-K is variable in humans; this burden is lowest in East Asian (EAS) individuals. Our study identifies population-specific sequence variation for HERV-K proviruses at several loci. We expect these resources will advance research on HERV-K contributions to human diseases.

## Author summary

Human Endogenous Retrovirus type K (HERV-K) is the youngest of retrovirus families in the human genome and is the only group of endogenous retroviruses that has polymorphic members; a locus containing a HERV-K can be occupied in one individual but empty in others. HERV-Ks could contribute to disease risk or pathogenesis but linking one of the known polymorphic HERV-K to a specific disease has been difficult. We develop an easy to use method that reveals the considerable variation existing among global populations in the prevalence of individual and co-occurring polymorphic HERV-K, and in the number of HERV-K that any individual has in their genome. Our study provides a reference of diversity for the currently known polymorphic HERV-K in global populations and tools needed to determine the profile of all known polymorphic HERV-K in the genome of any patient population.

## Introduction

Endogenous retroviruses (ERVs) are derived from infectious retroviruses that integrated into a host germ cell at some time in the evolutionary history of a species [1–5]. ERVs in humans (HERVs) comprise up to 8% of the genome and have contributed important functions to their host [6–8]. The infection events that resulted in the contemporary profile of HERVs occurred prior to emergence of modern humans so most HERVs are fixed in human populations and those of closely related primates. However some HERVs are still transcriptionally active and capable of causing new germline insertions so that individuals differ in the number and genomic location occupied by an ERV, a situation termed insertional polymorphism [9–11]. Among all families of HERVs, HERV-K is the only one known to be insertionally polymorphic in humans. However, HERV-K genomes are closely related and as with many repetitive elements, they are difficult to accurately assign to a genomic location using standard mapping approaches [12,13].

The DNA form of a retrovirus is called a provirus and minimally encodes the structural *gag* and *env* gene, and genes for a protease and polymerase, termed *pol*. Viral genes are flanked by long terminal repeats (5’ or 3’ LTR). While there are several HERV-K that are full length, none are infectious and most contain mutations or deletions that affect the open reading frames or truncate the virus. Further, the LTRs are substrates for homologous recombination, which deletes virus genes while retaining a single, or solo, LTR at the integration site [14–16]. Insertional polymorphism typically refers to the presence or absence of a retrovirus at a specific locus [17,18]. However an occupied site can contain a provirus in some individuals and a solo LTR in others and hence still display polymorphism. Thus HERV-K and other HERVs have contributed to genomic diversity in the global human population in several ways [19].

The presence of antibodies to HERV proteins or HERV transcripts has spurred a quest to determine if HERVs from multiple families have a role in either proliferative or degenerative diseases in humans [20–26]. Although there are known mechanisms by which a HERV can cause disease; for example, by inducing genome structural variation through recombination [27–31], affecting host gene expression [32], and inappropriate activation of an immune response by viral RNA or proteins [23], it has been difficult to establish an etiological role of a HERV in any disease. HERV-K specifically has been associated with breast and other cancers [3,33–37], and autoimmune diseases, such as rheumatoid arthritis [38,39], multiple sclerosis [22,40] and systemic lupus erythematosus [8,22,41] without definitive evidence of causality or of specific loci involved. Recently, a HERV-K envelope protein was shown to recapitulate the clinical and histological lesions characterizing Amyotropic Lateral Sclerosis [42,43], providing an important mechanistic advance of a role for a HERV-K protein in a disease. Despite growing evidence for a contribution of HERV-K transcripts or proteins to the pathogenesis of human disease, it is difficult to distinguish among HERV-K loci to investigate potential roles and, in particular, to determine if a loci that is polymorphic for presence or absence of a provirus could be involved.

In this paper, we focus on characterizing the genomic distribution of known insertionally polymorphic HERV-K proviruses in the 1000 Genomes Project (KGP) data. We present a data-mining tool and a statistical framework that accommodates low depth whole genome sequence data characteristic of the KGP—and often patient—data to estimate the presence or absence of a provirus at all loci currently known to contain a HERV-K provirus. Using these data, we determine the number of known polymorphic HERV-K proviruses per genome because HERV-Ks can affect genomic stability [44] contributing to the pathogenesis of a disease. We also provide a tool to visualize HERV-K co-occurrence in global populations to facilitate exploration of synergy that might exist among specific polymorphic HERV-K in disease [45]. Our results provide a reference of global population diversity in HERV-K proviruses at all currently known polymorphic loci in the human genome and demonstrate that there are notable differences in the prevalence of HERV-Ks in different global populations and in the total number of HERV-Ks currently known to be polymorphic within a person’s genome.

## Results

### A model to estimate polymorphic HERV-K from whole genome sequence data

The goal of this research was to develop a computationally efficient and easy to use tool that could accurately report the status of all reported insertionally polymorphic HERV-Ks with coding potential (provirus) from whole genome sequence (WGS) data. We use the KGP database, which represents individuals in five super-populations and 26 populations, to establish the diversity in global populations at each known polymorphic HERV-K proviral locus and the total number of these polymorphic HERV-K in individual genomes to provide a foundation to study the role of HERV-K in human disease. Our reference set consists of all HERV-K sequences that are available in public databases and that can be unambiguously assigned a location in hg19. Sequences of HERV-K that are not present in hg19 but that were generated by PCR primers to the host flanking regions are included in the reference HERV-K set. From these HERV-K reference sequences, we generate a set of *k-mers* (see S2 Fig for optimizing k) that are unique to all HERV-Ks at each locus. The analysis of subject data starts with a data mining step that recovers all whole genome sequence reads that map to identified HERV-K elements in hg19. The rationale here is that polymorphic HERV-K that are not present in hg19 are greater than 80% homologous to those in the human reference genome and will map on existing elements. The recovered reads from a query WGS data set are then reduced to *k-mers* and mapped, requiring 100% match, to the reference set of *k-mers* (T), which represents all unique sites for HERV-K at each locus. The output is a ratio (n/T) of subject *k-mers* (n) that are 100% match to the reference *k-mers* (T) (see Methods for full details; the value of T for each HERV-K is in S1 Dataset:virus).

**Fig 1:**
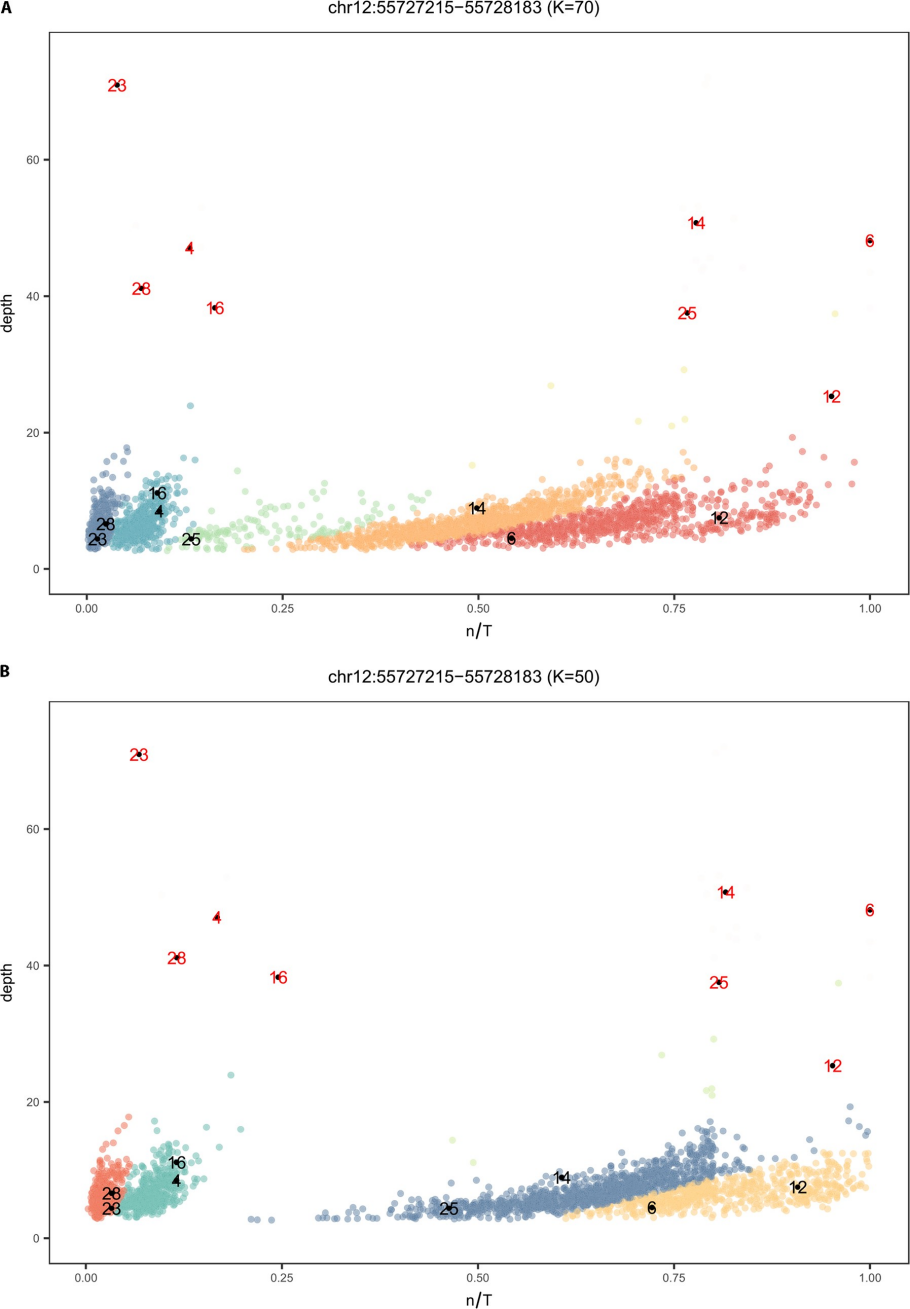
A mixture model to account for low depth WGS data. A) Mixture model output on n/T values of 2535 individuals from KGP with low depth sequence data for chr12:55727215–55728183 when K = 70. At this value of k there are clusters representing low n/T values that are not well resolved and individual 25 and 14, which have the same status in high depth data, are assigned to different clusters. B) The result of the mixture model on the same data with k optimized to 50. The model returns four clusters each indicated by a unique color and eight of the 28 individuals that have both low and high depth sequence data are shown (see S1 Dataset:KGP for identification). The n/T ratio is 1 for persons with high depth data [red numbers, #6 and 12] who have the reference allele, while the corresponding low depth data [black numbers, yellow cluster] from the same individuals have n/T ranging from 0.7 to 0.9. There is less of an effect of sequence depth for individuals who do not have the HERV-K (n/T = 0, red cluster, #23 and 28). However optimizing k improves separation of the solo LTR (green cluster; #4 and #16) from the blue cluster (#25 and #14), which represents a state where some unique k-mers in the set T are missing in the query data (this is likely an allele; see S3 Fig). States are confirmed by mapping the *k-mer*s from individuals in a cluster to the reference HERV-K at this locus (S3 Fig).

Our preliminary analysis of the KGP data demonstrated that our *k-mer*-based approach is sensitive to sequence depth; some HERV-K loci are represented by an almost continuous range of n/T values from 0–1 (S1 Fig), making presence/absence classification difficult. However, the majority of the KGP data is approximately 6x depth and thus to make use of this important resource, we developed a mixture model to statistically assign the n/T values from genomes to a cluster considering the sequence depth. K was optimized to 50 because this value improved our model computational efficiency and output (Fig 1B, S1 Text, S2 Fig). The affect of sequence depth on n/T can be seen by comparing the sequence data of 28 individuals in the KGP data that have both low and high sequence depth data (Fig 1 shows a subset of eight individuals for clarity). If read depth is greater than 20, there is less dispersion of n/T values, most likely because more reads from the query WGS data are recovered from the mapped intervals. The states, ‘provirus’, ‘solo LTR’, and ‘absent’ are preliminarily assigned to each cluster based on the high depth data (data in Fig 1B used for description below). Individuals with n/T = 1 have the reference allele (represented by the yellow cluster of low depth data) and n/T = 0 (red cluster) indicates that the HERV-K is absent (no *k-mer*s to unique sites in the HERV-K at this locus were recovered from mapped sequence reads). The *k-mer*s derived from persons with low (green) and intermediate (blue) n/T values were mapped to the HERV-K reference for this locus to determine whether they localized only in the LTR (assign ‘solo LTR’ to green cluster) or in the coding region (assign ‘provirus’ to blue cluster) (S3 Fig).

### Prevalence of polymorphic HERV-K in each KGP super-population

The WGS data of each individual in the KGP dataset were evaluated using our optimized analysis workflow. HERV-Ks on chrY were not considered. Twenty sites, omitting one at chr1:73594980 [see Methods] that have been reported to be polymorphic for presence/absence [10,11,34,46] were identified as polymorphic for a HERV-K provirus by our analysis (S1 Dataset:virus). Polymorphic HERV-Ks greater than 6 kbp in length cluster together in a phylogenetic analysis indicating that they are closely related (S4 Fig). The prevalence (proportion of individuals in a given population with a provirus present at a given locus) of the 20 polymorphic HERV-K proviruses varied from 0.9% to 99.5% when averaged across the entire KGP dataset (Table 1). However, there were notable differences in prevalence at each HERV-K site among the five super-populations (AFR, EAS, AMR, EUR, SAS; see Methods for key to abbreviations). Of the 20, the prevalence of seven polymorphic HERV-Ks was greater than 90% and the difference between populations with the lowest and highest prevalence was less than 6.5% (Table 1). There was 100% occupancy for six of the seven high prevalence polymorphic HERV-Ks (98.8% for the seventh), indicating that the rate of conversion to solo LTR is low for viruses at these sites (see S1 Text for occupancy and S2 Dataset:KGP(absence, solo, presence) for model prediction of solo LTR prevalence). Two polymorphic HERV-Ks had an overall prevalence of less than 10% in any population (Table 1) and were found in individuals of AFR origin; we found no evidence of a solo LTR at these two sites. Nine of the remaining 11 HERV-Ks are of interest because the difference between super-populations with the highest and lowest prevalence is between 28 and 80 percentage points (Table 1). Of note, for the three HERV-Ks with the largest difference among super-populations, the prevalence is lowest in EAS populations.

**Table 1:** Provirus frequencies of polymorphic HERV-K.

	KGP	AFR	AMR	EAS	EUR	SAS
**chr1:75842771**^**c**^	42.88	26.76	56.53	6.02	68.91	66.80
**chr3:112743479**^**a**^	98.46	96.71	99.72	99.81	99.60	97.37
**chr3:148281477**	41.89	38.86	42.61	45.05	46.53	37.45
**chr3:185280336**^**a**^	99.49	98.06	100.00	100.00	100.00	100.00
**chr4:69463709**^**c**^	72.50	93.87	88.92	31.07	85.35	61.94
**chr5:156084717**^**a**^	99.41	98.36	99.72	100.00	99.80	99.60
**chr6:57623896**^**a**^	93.65	90.73	97.16	90.87	97.23	94.33
**chr6:78427019**^**a**^	97.71	95.52	97.16	99.61	97.23	99.60
chr7:4622057*^c^	47.50	61.14	30.11	58.25	36.44	41.50
**chr8:12316492**^**c**^	14.08	32.88	12.22	0	15.64	3.04
**chr8:7355397**^**c**^	18.66	39.16	12.50	6.02	11.29	15.99
**chr10:27182399**^**a**^	99.13	97.46	99.43	99.81	99.80	99.80
**chr11:101565794**^**c**^	63.04	80.87	77.27	6.99	86.53	63.16
**chr12:55727215**	72.19	72.80	80.40	63.30	80.99	65.79
**chr12:58721242**^**c**^	70.73	58.89	78.41	60.00	87.33	75.51
**chr19:21841536**^**c**^	26.98	39.16	11.93	32.23	10.69	32.39
**chr19:22414379**^**c**^	67.77	89.24	60.80	56.89	55.84	67.21
**chr19:22457244**^**b**^	0.87	3.29	0.00	0.00	0.00	0.00
**chr22:18926187**^**a**^	99.49	98.36	99.72	100.00	99.80	100.00
**chrX:93606603**^**b**^	2.25	7.32	2.27	0.00	0.00	0.00

For simplicity, only the starting coordinate is listed.

* The value given represents individuals containing the tandem repeat found in hg19

^a^: prevalence > 90%

^b^: low prevalence HERV-K and no individuals with only a solo LTR

^c^: max-min difference is > 28%

underline: AFR significantly different from other 4 super populations.

See S2 Dataset:compare_prevalence for full analysis of the data.

Individuals from African populations differ significantly from the other four super-populations in the prevalence of ten of the polymorphic HERV-K, three of which occur in close proximity on chr19. (Table 1, S2 Dataset:compare_prevalence). EUR and AFR super-populations are significantly different in the prevalence at all but one of the 20 polymorphic HERV-K based on adjusted p-values (S2 Dataset:compare_prevalence).

### The number of polymorphic HERV-Ks per individual

**Fig 2:**
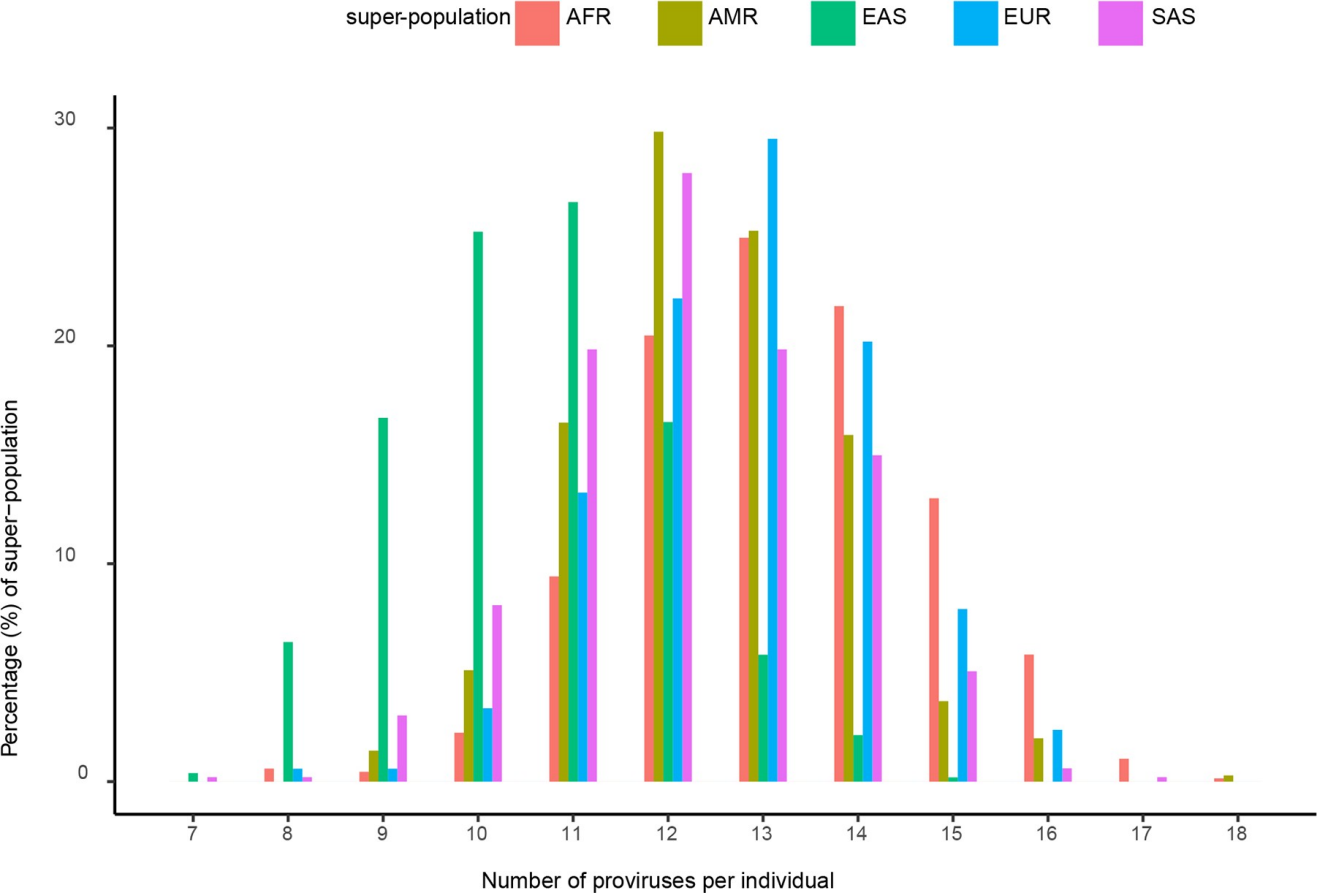
Histogram of the number of proviruses per individual from the KGP. The number of the 20 known polymorphic HERV-K proviruses in individual from each of the five KGP super-populations, represented by indicated colors.

The HERV-K genome is close to 10 kbp. As there are 20 known HERV-K loci with the potential to encode a provirus that are polymorphic in human populations, we asked if there is a difference in the burden of these repetitive, and potentially functional, viral elements among individuals. This was indeed the case. Of the 20 polymorphic HERV-K proviruses assessed, the number per person’s genome ranges from 7–18 (Fig 2, S2 Dataset:HERV-K per person). More than 63% of individuals from all super-populations except EAS carry 12 to 14 proviruses in their genome. Individuals from EAS have a lower burden with 69% of individuals carrying 9–11 of the 20 polymorphic HERV-K proviruses. 7% of AFR individuals have 16 or 17 proviruses compared to a maximum of 2% in other groups (S2 Dataset:HERV-K per person). These data suggest that a comprehensive investigation of polymorphic HERV-Ks may be a more productive means to advance studies of their potential disease impact.

### Co-occurrence of polymorphic HERV-Ks

**Fig 3:**
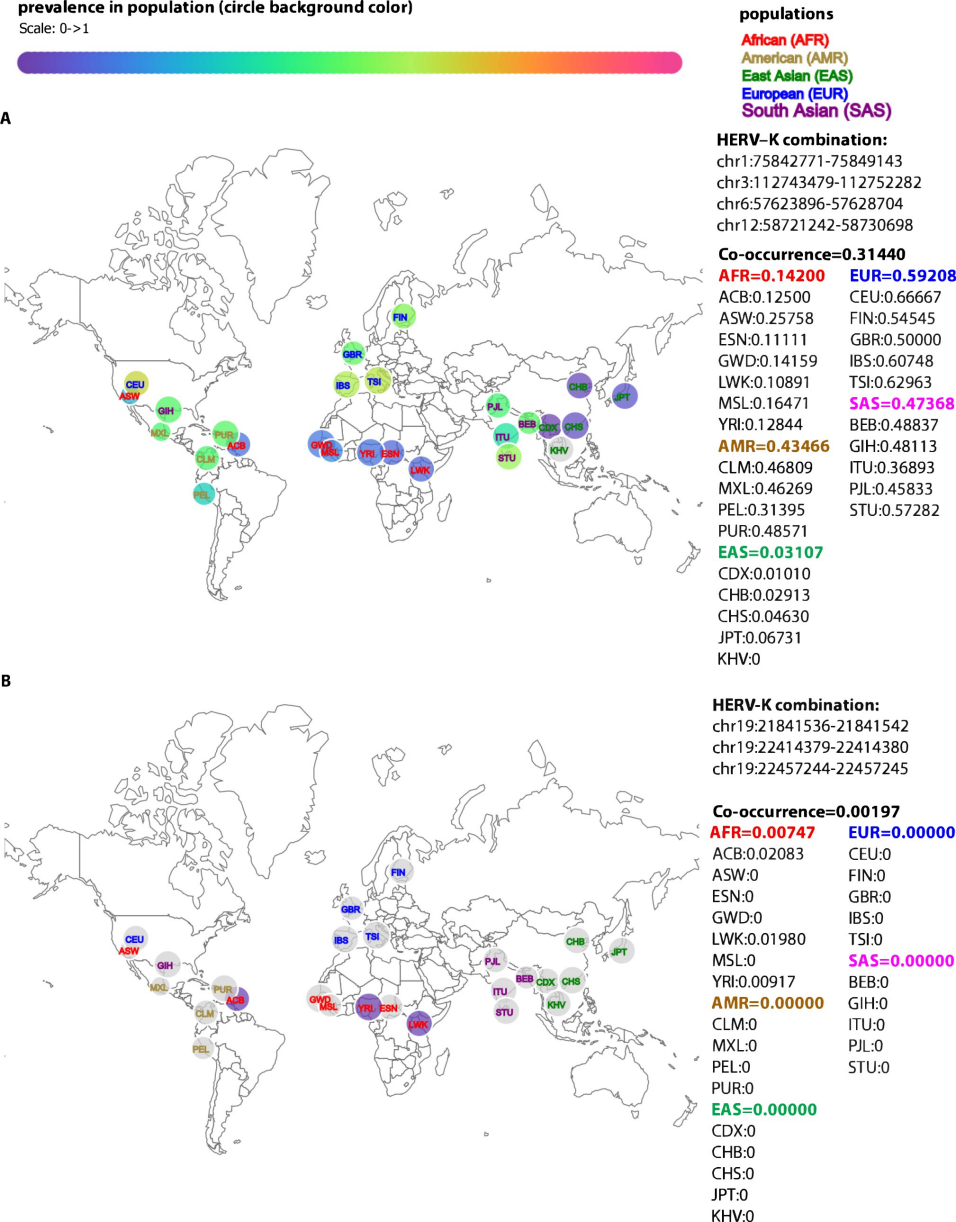
A visualization tool to examine co-occurrence of polymorphic HERV-Ks. A) The co-occurrence of polymorphic HERV-Ks at chr1:75842771–75849143, chr3:112743479–112752282, chr6:57623896–57628704, and chr12:58721242–58730698 in the 26 populations are represented based on their geographic location. The relative prevalence for these four co-occurring HERV-Ks in each population bubble is displayed based on the color gradient shown in the scale at the top. The actual prevalence of the given combination of HERV-K provirus for each population and the cumulative prevalence for each super-population are shown in text on the right. Note that AFR and EAS have the lowest prevalence of these four polymorphic HERV-Ks. B) As in (A) showing the co-occurrence of the three polymorphic HERV-Ks that are present on chr19 by population. This is a rare combination only found in two AFR populations and individuals in the Caribbean of African ancestry.

Our data provide a comprehensive picture of sites occupied by HERV-K provirus in each genome. Although most previous studies investigating a role of HERV-K in human disease assessed the prevalence of the HERV-K at a given locus, it is possible that, for example, two HERV-Ks each at 40% prevalence in a population rarely co-occur in an individual genome. By providing the status of all known polymorphic HERV-K in the genome, our tools facilitate such assessment and can advance investigation of HERV-K and human disease. We assessed combinations of three, four and five polymorphic HERV-Ks in KGP data and found that there are many combinations of co-occurring viruses that are population-specific (S3 Dataset). To facilitate exploration of HERV-K combinations among KGP populations, we developed a D3.j visualization tool (see Methods) that allows a user to choose any combination of the 20 polymorphic HERV-K proviruses and display the co-occurrence prevalence among the 26 populations represented in the KGP data. As an example, we show a combination of four HERV-Ks to represent the variation that occurs in KGP individuals, which in this case ranges from 3% in EAS to 59% in EUR (Fig 3A). We also determine that the three polymorphic HERV-Ks found on chr19 co-occur only from three AFR populations and in less than 2% of individuals (Fig 3B).

### KGP super-populations are distinguished by HERV-K status

**Fig 4:**
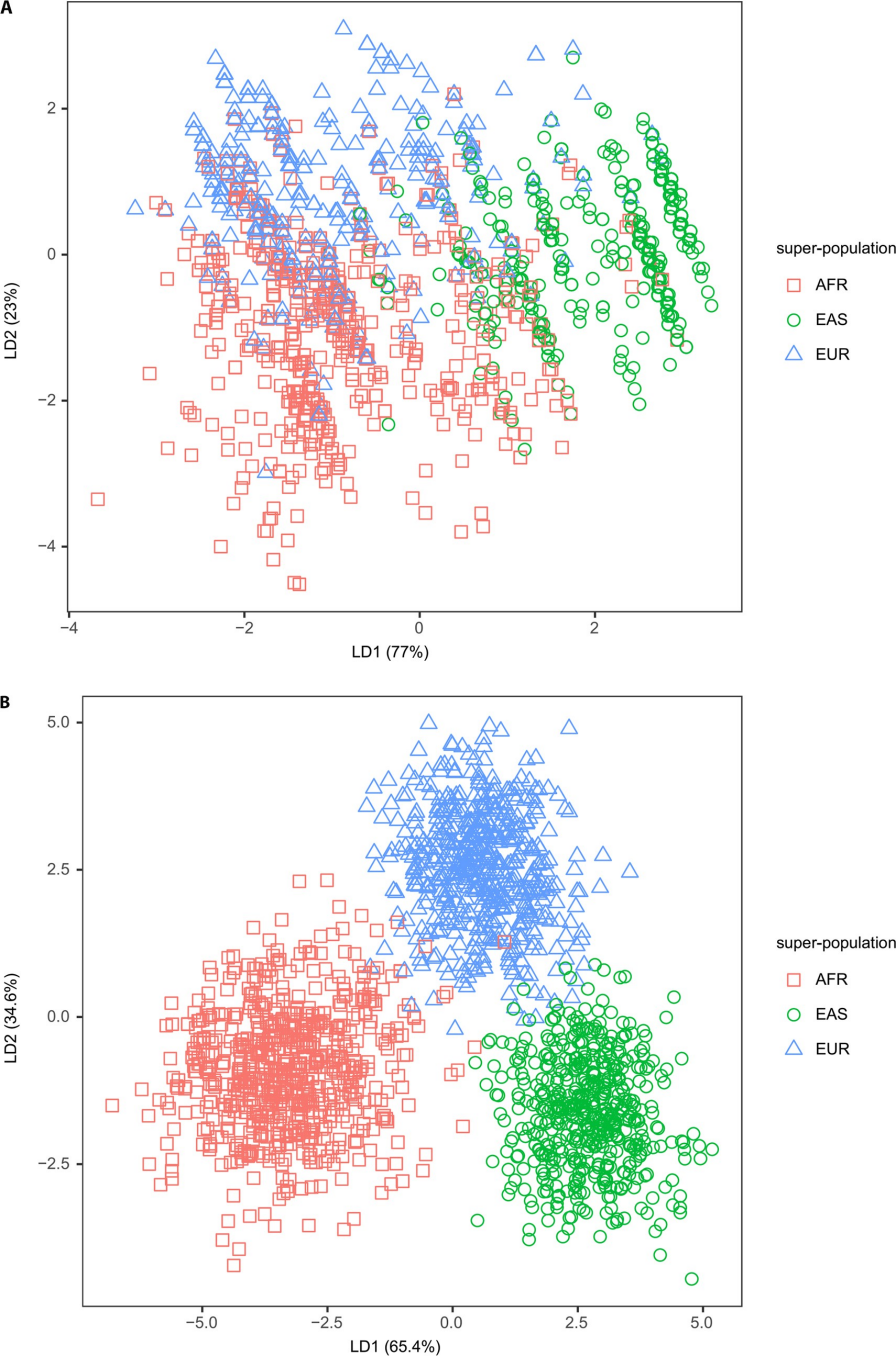
Linear discriminant analysis of HERV-K status among three super-populations. A) LDA based on the states ‘provirus’, ‘solo LTR’ and ‘absence’ of the 20 polymorphic HERV-K for AFR, EAS, and EUR. AMR and SAS overlap these three populations and are removed for clarity B) LDA plot on n/T ratio of all 96 HERV-K separates AFR, EAS, and EUR super-populations. See S6 Fig for plots with all five super-populations.

Because there are clearly population-specific differences in both individual HERV-K prevalence and in the prevalence of HERV-K co-occurrence, we explored whether the presence or absence of these 20 documented polymorphic HERV-Ks is sufficient to distinguish populations using Fisher’s linear discriminant analysis (LDA) [47]. Based on the status ‘provirus’, ‘solo LTR’, or ‘absence’, there is little resolution of AFR, EUR, and EAS super-populations (Fig 4A). However, there is sufficient signature to separate AFR, EUR, and EAS if we utilize the n/T ratio of the 20 polymorphic HERV-Ks (S5 Fig) and we further improve population separation if we use the n/T ratio for all 96 HERV-Ks (Fig 4B). This indicates that we are losing information by reducing the data to three states and that fixed HERV-K also contain signal for population of origin.

**Fig 5:**
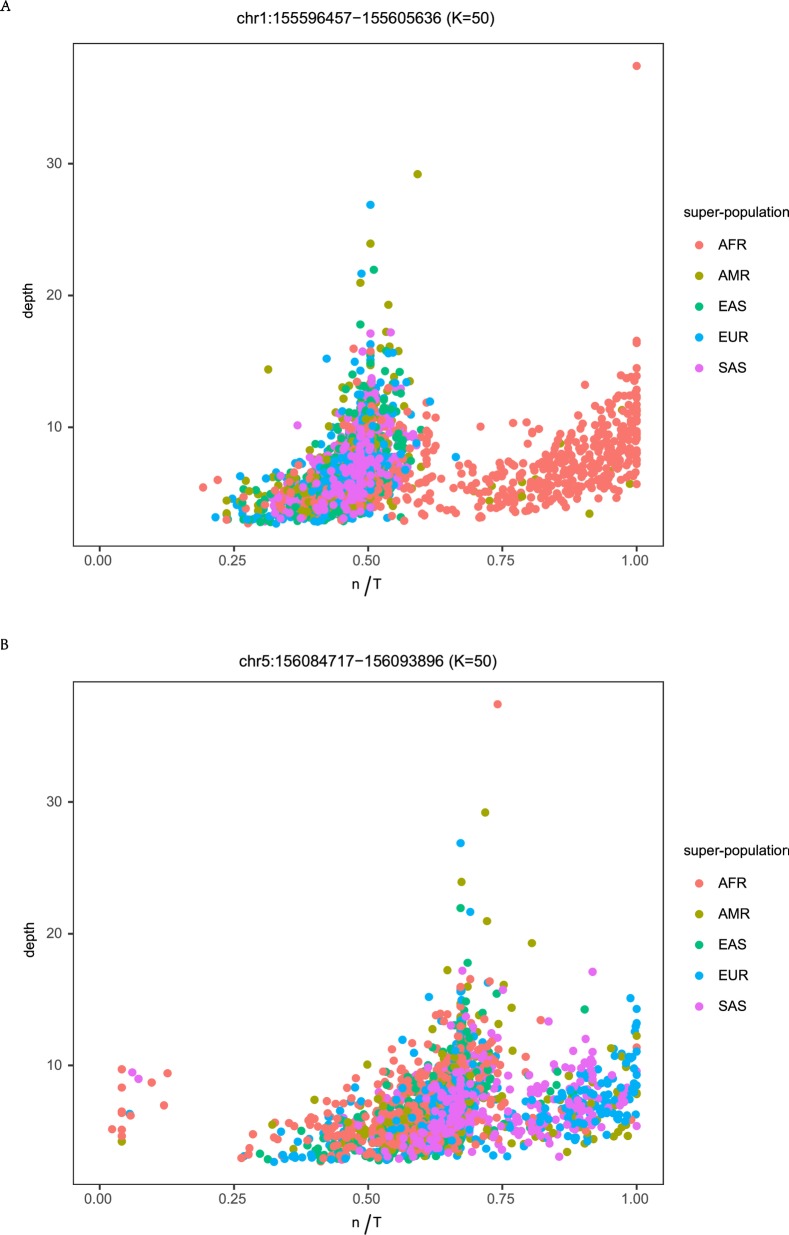
Population specificity of HERV-K alleles. A) n/T plot for HERV-K at chr1:155596457–155605636 colored by each of the 5 super-populations. Only individuals from AFR and a few from AMR have an n/T approximating 1 indicative of the HERV-K reference sequence. B) Plot of chr5:156084717–156093896 colored by each of the 5 super-populations. In this case, all populations except AFR have the reference allele and all super-populations have an alternative allele that is not present in our reference set.

An n/T = 1 indicates that the query set contains all *k-mers* that map to the reference set T for a specific HERV-K. If there is a HERV-K allele that has not been reported in any database but that is common in a population, we expect n/T <1 because we require 100% match to reference set T and *k-mers* covering allelic sites will be excluded (see Fig 1B, blue cluster for an example). We assessed the density distributions of n/T plots for each of the 96 HERV-Ks for evidence of population-specific alleles (S1 Text, S7 Fig). Five HERV-Ks have some indication of population specific distributions (S1 Dataset:virus). The HERV-K at chr1:155596457–155605636, which we report as fixed, is notable because the reference allele (n/T = 1) is only found in AFR (Fig 5A, S7 Fig). Individuals from other populations have n/T near 0.5. We mapped *k-mer*s from individuals with n/T near 0.5 to the reference HERV-K sequences and confirmed that there is a loss of *k-mer*s at several sites covered by the unique reference *k-mer*s for this virus (S8 Fig). There are also cases where the reference allele is found in all populations except AFR (Fig 5B and see S7 Fig for additional examples).

## Discussion

Our research provides a tool to mine whole genome sequence data to collectively evaluate the status of HERV-K provirus at known polymorphic and fixed sites in the human genome. The tool incorporates a statistical clustering algorithm to accommodate low depth sequence data and a visualization tool to explore the co-occurrence of known polymorphic HERV-K in the global populations represented in the KGP data. There are numerous significant differences in the prevalence of individual and co-occurring known polymorphic HERV-K among the five KGP super-populations. It is notable that individuals from EAS carry a lower total burden of the 20 polymorphic HERV-K than other represented populations. These data provide a comprehensive framework of genomic diversity among 20 documented polymorphic HERV-K proviruses to advance studies on potential roles for HERV-K in human disease, which have been alluring yet difficult to establish [21,22,24].

Tools developed to interrogate ERV insertional polymorphism typically exploit the unique signature created by the host-virus junction [11,48,49]. These approaches indicate that a site is occupied by an ERV but not whether there is a provirus associated with the site, which is more difficult to accomplish with short read sequence data. Our analysis tool provides an efficient means to detect occupancy and provirus status in one step. We decrease computational time by analyzing only the set of reads that map to existing HERV-K loci in the reference genome. This approach is justified because the known polymorphic HERV-K that are missing from the human reference are closely related to those in the reference genome assembly (see S4 Fig) and hence reads derived from them map to a related HERV-K in the reference. We employ *k-mer* counting methods, which also increase computational efficiency. A reference set of *k-mer*s that is unique to each HERV-K is generated for each location in the genome and the proportion of reads (n/T) from the query set that maps to the *k-mer* reference set is reported as a continuous variable; there is no threshold of read count or coverage imposed for classification. Instead we utilize a mixture model to statistically cluster values based on n/T and sequence depth and assign the same HERV-K status to all individuals in a cluster. Clusters representing n/T of 1 consist of individuals from whom all the unique *k-mer*s identified in the HERV-K reference set were recovered from their mapped WGS data. We classify other clusters by determining if *k-mer*s mapped on the reference allele are distributed at sites in the coding portion of the genome or only in the LTR; reads mapping only in the LTRs are classified as solo LTR. This approach demonstrated that the *k-mers* derived from some individuals only covered a subset of the unique sites and led to the interesting finding that several HERV-K loci could have population specific alleles.

Wildschutte *et al* [11] have conducted the most comprehensive study of HERV-K prevalence in the KGP data to date. The goal of that paper was to identify new polymorphic insertions, either provirus or solo LTR, based on detecting reads containing the host virus junction. However, they implemented an additional step to detect provirus and provide the prevalence of some polymorphic HERV-K provirus for comparison with our results (see S1 Dataset:virus for comparison of prevalence values reported in Wildschutte *et al* [11]). There are five HERV-K previously reported in Subramanian *et al* 2011 [10] that were not included in Wildschutte *et al* [11]; all are polymorphic in our analysis (range 43–99%, see Table 1 and S1 Dataset:virus-column N). Seven polymorphic HERV-K, which Wildschutte *et al* [11] indicate occur in greater than 98% of KGP individuals, are fixed in our study. Our estimated prevalence for 14 HERV-K differs from that reported in Wildschutte *et al* [11] by 5% or more. Of these 14, the prevalence estimates at chr1:155596457–155605636 are most divergent. Our data show this site is fixed for provirus and Wildschutte *et al* [11] report that only 14% of the KGP data, all from AFR, have a HERV-K provirus integration. Our plots for chr1:155596457–155605636 show that AFR individuals carry the reference allele at this site (n/T near 1, Fig 5A) and all other individuals have n/T near 0.5. The *k-mer*s from individuals with low n/T values for chr1:155596457–155605636 map to only a subset of sites marked by unique *k-mer*s in the coding region (S8 Fig), which is consistent with sequence polymorphism or a deletion at these positions. The reference set T is small for this HERV-K and therefore overall coverage of the genome is low. Because Wildschutte *et al* [11] used a minimum coverage threshold for their *k-mer* mapping method, it is possible that alleles present in non-AFR populations do not meet their inclusion criteria. There is a similar signal for alleles, represented by lower n/T values, at the other 13 HERV-K sites although the differences between our prevalence estimates and those of Wildschutte *et al* [11] are small (S1 Dataset:virus). In most cases these putative alleles are found in all populations at different frequencies but in five there is some degree of population specificity (Fig 5, S7 Fig, S1 Dataset:virus). Our results indicate that there could be considerably more sequence variation in HERV-K among human populations than previously appreciated. These data also suggest that using a HERV-K consensus sequence to study pathogenic potential could miss important features of HERV-K proviral polymorphism, which can be characterized by both the site occupancy status (presence/absence) and, when present, by sequence differences among individuals.

HERV-Ks are the youngest family of endogenous retroviruses in humans and consequently they share considerable sequence identity. This has the effect of limiting the number of unique sites associated with some HERV-K, which decreases the size of the reference set T (S1 Dataset:virus). The set T is small for near identical HERV-K such as HERV-Ks involved in a duplication event. The HERV-Ks at chr1:13458305–13467826 and chr1:13678850–13688242 are identical and cannot be distinguished. We report n/T for only one of these HERV-K (see S1 Dataset:virus, column M). We treat the two HERV-K proviruses spanning chr7:4622057–4640031 as a single virus with n/T = 1 reflecting the tandem arrangement found in the hg19. In this case, n/T<1 can mean either that both proviruses are present but with substitutions at a unique *k-mer* site or that one provirus converted to a solo LTR. Thus although an n/T ratio of 0 or 1 reliably indicates absence and presence of reference HERV-Ks, respectively, when T is small, sequence polymorphism and a deletion event can be difficult to distinguish from a solo LTR. However, because our mixture model statistically clusters similar n/T values based on sequence depth, all individuals in a cluster have the same status (e.g allele or solo LTR) even if we do not know what that state is. The ability of our tools to resolve the status of closely related HERV-K provirus sequences will improve as more empirical sequence data becomes available.

Our approach provides researchers with a rapid means to determine if the prevalence, and overall burden of the 96 HERV-K proviruses evaluated differ between a patient data set and the population represented in KGP to which they trace ancestry. The visualization tool will facilitate investigation of combinations of HERV-Ks in certain clinical conditions. The potential that HERV-K has multiple allelic forms in different populations is worthy of further analysis because a sequence allele could also contribute to a disease condition.

## Materials and methods

### HERV-K proviruses

The 96 HERV-K proviruses previously reported [10,11,34,46] were supplemented with HERV-K alleles present in the NCBI nt database (November 2016 release) (92 in hg19, and 4 from the NCBI nt database). We required that any allele of a HERV-K from the nt database have at least 2kb of hg19 reference-matching host flanking sequence to confirm genome location. In total, 234 alleles were collected at the 96 known HERV-K loci. The location information and virus features are summarized in S1 Dataset: virus.

### Developing a *k-mer* based detection model

We identified the *k-mer*s that correspond to unique sequence characterizing each HERV-K. *K-mer*s are substrings (subsequences) of length *k* that exist in a string (DNA sequence). The length *k* is determined empirically (S1 Text). Each *k-mer* is labeled with the corresponding viruses in which it is observed.

Only those *k-mer*s referring to a single virus locus, unique *k-mer*s, are selected for the set T. Where multiple alleles of a HERV-K are available, *k-mer*s unique to all alleles at that location comprise T. Multiple 2bps different *k-mer*s (such as SNPs) corresponding to the same location on the virus, are merged into a single entry for the purposes of computing T. We map unique *k-mer*s back to the corresponding alleles to determine coverage of the HERV-K and whether *k-mer*s are located in LTRs (S3 Fig; S1 Dataset: virus).

### Analysis of 1000 genome project (KGP) data

To develop a method to recover sequences containing information on HERV-K we leverage the fact that HERV-Ks are closely related. Thus, most sequence reads obtained from an individual with a polymorphic HERV-K that is absent in the human reference, hg19, will map to the location of a closely related HERV-K that is present the human genome reference. (As we show in S4 Fig, the known polymorphic HERV-K proviruses are closely related.) A file with the coordinates for all reported HERV-K insertions is used to extract mapped reads from a genome sequence file (S1 Dataset:bed, which provides the coordinates for both hg19 and hg38). Note that the KGP data were mapped to GRCh37, which includes the decoy sequence hs37d5. This decoy contains the HERV-K at chr1:73594980_73595948, which is not present in hg19. Thus, we did not recover any reads for this HERV-K, which is polymorphic but reportedly at high prevalence in most populations [11].

The KGP data were downloaded in aligned Binary Alignment/Map (BAM) format (ftp://ftp.ncbi.nlm.nih.gov/1000genomes/ftp/data/). It contains data for 2,535 individuals (S1 Dataset:KGP) sequenced via low-depth whole-genome sequencing (mean depth = 6.98X). The individuals represent 26 populations, derived from 5 super-populations, including African (AFR), Admixed America (AMR), East Asian (EAS), European (EUR), and South Asian (SAS) [50,51]. Of 2,535 individuals, 28 also have high-depth DNA sequences (mean depth = 48.06X), which we use as a pilot dataset to develop the mixture model, described below and in Supplementary Text.

Our computational framework to indicate the status of each known HERV-K provirus is based on the n/T ratio, which is the proportion of *k-mer*s in the data mined from WGS of each individual that are identical to the reference set T for each HERV-K provirus. Sequence reads are extracted from a mapped file of whole human genome sequence data based on coordinates corresponding to each annotated HERV-K. The reads are k-merized and mapped to the set T, which represents all unique *k-mer*s assigned to each HERV-K in the reference set. We use exact match to map the *k-mer* data set to the unique *k-mer* references. The n/T ratio is an indicator of the presence of each HERV-K; n/T = 1 indicates that the individual has the HERV-K in our reference dataset documented to be at that locus while n/T = 0 indicates that no *k-mers* unique to a HERV-K locus were recovered (see Fig 1 for more explanation). Using a hash table (S1 Text), it takes 15 minutes to generate the n/T matrix for 100 files. The source code for the entire process is at https://github.com/lwl1112/polymorphicHERV

### Dirichlet process Gaussian mixture model (DPGMM)

We utilized a statistical model to account for the dependency of the number of *k-mer*s obtained from a person’s sequence data (denoted by *n*_*ik*_ for the *i*th subject and *k*th HERV-K, with *i* = 1,…,*I*,*k* = 1,…,96) that maps to the reference set T for each HERV-K on sequencing depth. Thus for each HERV-K we could statistically cluster those *n*_*ik*_/*T* values for *i* = 1,…,*I* based on the sequence depth of the WGS data for each individual for subsequent biological classification (provirus, solo LTR, absence, see Fig 1). More specifically in our analysis, for each *k* HERV-K, *k* = 1,…,96, consider a sample of size *I* measurements *x*_i_ (*i* = 1:*I*), where each *x*_i_ is a vector of length 2 *x*_*i*_ = (*x*_i1_,*x*_i2_) with *x*_i1_ being the *n*_*ik*_/*T* measurement and *x*_i2_ the log function of depth. Here, for notation simplification, we use *x*_i_ instead of *x*_ik_. To perform clustering analysis, we utilize the mixture model approach, which is arguably the most widely used statistical method for clustering. Specifically, we follow the work proposed by Lin et al. [52] that employs a Gaussian Mixture Model (GMM) with density function given by

$$f\left(x_{i}|\theta \right)=\sum_{j=1}^{M}\pi_{j}\;N\left(\mu_{j},\Sigma_{j}\right),\;for\;i=1:I$$ (1)

where all relevant and needed (unknown) parameters are represented by *θ* = (π_{1:M},_ μ_{1:M}_, *Σ*_{1:M}_). *N*(μ_*j*_,*Σ*_*j*_) is the Gaussian density for the jth component parameterized by the 2-dimensional mean vector μ_*j*_ and 2x2 covariance matrix *Σ*_*j*_. π_{1:M}_ are the mixture components prior probabilities summing to 1. To allow a flexible modeling approach, we employ the standard Bayesian (truncated) Dirichlet Process prior for the parameters *θ* = (*π*_*j*_, μ_*j*_, *Σ*_*j*_, *j* = 1:*M*) [53,54]. The idea is that some of the mixture probabilities (*π*_*j*_) can be zero, hence the actual number of mixture components needed may be smaller than the upper bound M. This mechanism allows automatic determination of the number of mixture components needed by the data set at hand. For model estimation, a latent indicator *Z*_*i*_ ∈{1,2,…,*M*} with *P*(*Z*_*i*_ = *j*) = *π*_*j*_ is used, for *i* = 1:*I*. Specifically, *Z*_*i*_ = *j* if, and only if, *x*_i_ comes from component *j*. Given a fitted model via the Bayesian expectation–maximization algorithm, in terms of estimates of all parameters *θ*, instead of interpreting the fitted Gaussian mixture components as clusters, we identify clusters by aggregating Gaussian components so that non-Gaussian type of clusters can be flexibly represented. Merging components into clusters can be done by associating each of the Gaussian components to the closest mode of *f*(*x*_1:*I*_|*θ*) = ∏_*i* = 1:*I*_*f*(*x*_*i*_|*θ*). Hence, the number of modes identified is the realized number of clusters. [S1 Text for additional detail]

### Co-occurrence of polymorphic HERV-K

We consider that both the individual prevalence of a HERV-K and the co-occurrence of multiple HERV-Ks could differ among populations.

The time of a brute-force approach for finding all combinations *C*_*m*_ of size m from p polymorphic HERV-K is (∑m=1p(pm)=2p−1), which is not efficient and is redundant. We employed the Apriori algorithm [55], which is commonly used for finding frequent pattern sets; in our case indicating which of the known polymorphic HERV-K frequently appear together. It first generates combinations C_m_ (initialized to 1). In the optimization, frequent combinations F_m_ are returned from candidates C_m_ when prevalence exceeds the minimum threshold of co-occurrence. F_m_ are then self-joined to generate combinations C_m+1_ of size *m* +1 and out of which F_m+1_ satisfy the minimum co-occurrence. In each pass, candidate combinations are pruned so as to avoid generating all combinations, which reduces running time significantly.

### Statistical analysis of HERV-K frequencies across populations

We made statistical comparisons across 5 super-populations for the following three problems. For each problem, there are (52) = 10 families of 1-to-1 comparisons conducted. The ‘prop-test’ function in R is used to test whether the proportions for two super-populations are the same.

1. individual prevalence of polymorphic HERV-K. (20 comparisons for each polymorphic HERV-K in a family)
2. The number of polymorphic HERV-K present per individual. (21 comparisons as the number of co-occurring polymorphic HERV-K is from 0 to 20)
3. The co-occurrence for combinations of polymorphic HERV-K.

Therefore, multiple hypotheses would be conducted on frequencies *F* across super-populations *P*_1…5_ as follows:

Null hypothesis, $H_{0}:F_{P_{i}}=F_{P_{j}}$, where i≠j;

Alternative hypothesis, $H_{A}:F_{P_{i}}\neq F_{P_{j}}$, where i≠j.

A separate P-value is computed for each test and the Benjamini-Hochberg procedure [56] is used to account for multiple comparisons.

### Visualization in D3.js

We utilized D3.js (Data Driven Documents) [57], an open-source java script library to create an interactive visualization to display co-occurrence of polymorphic HERV-Ks in human populations. Our visualization system includes two modules, a welcome page and a result page. Input JSON data include locations of polymorphic HERV-K, population information, and the 0/1 (absence / presence) matrix. (See S1 Text). Source code is available at: https://github.com/lwl1112/polymorphicHERV/tree/master/visualization and a searchable tool with the data reported here is at: http://pages.iu.edu/~wli6/visualization/

## Supporting information

S1 Text This file contains methods, table of site occupancy, and references cited in methods. (DOCX)

S1 Fig The distribution of n/T values for chr12:55727215–55728183 when k = 70. The x-axis is the n/T ratio, representing the proportion of k-mers derived from an individual’s genome data that matches the unique set T for the HERV-K at chr12:55727215–55728183. The y- axis represents sequence depth. Under these conditions, there is a tendency for clustering of some values but dispersion of points is broad and separation into biologically meaningful clusters would be difficult. For this reason, we developed the mixture model after optimizing the length k to facilitate clustering (S2 Fig and S1 Text).
(TIFF)

S2 Fig Effect of *k* on n/T. Six individuals with both high and low depth data are used to demonstrate how varying the length of k affects n/T values for absent, solo LTR and present states. High depth data is above the line (depth = 20). Different colors represent different values of *k* from 30–70 as shown in the legend. Each number represents a different individual (see S1 Dataset:KGP for the identify of the sample corresponding to each number).
(TIF)

S3 Fig Alignment of unique k-mers to HERV-K at chr12: 55727215. All k-mers derived from the data mining step from each individual are mapped to the reference set of unique k-mers, T, requiring 100% identity, to generate the set ‘n’ The first row shows the coverage of the set *T* on the HERV-K. The following plots show the mapping of the k-mer set ‘n’ from 8 individuals for the HERV-K at chr12: 55727215. # 6, 12, 14, and 25 (see S1 Dataset: KGP, column D for identification information) are labeled as ‘provirus’. Note the drop out of the peaks near 3500 and 5000bp for #14 and #25, which accounts for a decrease in n/T in these individuals. #4 and 16 have low n/T and k-mers map to the LTR region indicated above the diagram; these are labeled as ‘solo LTR’. #23, and 28 are labeled as ‘absent’. For individuals with states ‘solo LTR’ and ‘absent’, there are some peaks in the coding region. This is most likely the result of assigning unique k-mers to this HERV-K that are shared with those from a HERV-K that is absent from the reference HERV-K dataset.
(TIF)

S4 Fig Maximum likelihood phylogenetic tree of fixed and polymorphic HERV-K. To improve the alignment, only > = 6,500 bp HERV-Ks were included except for the HERV-K at chr1:75,842,771, which has a long deletion but aligns well in other regions. Maximum likelihood tree was generated using PhyML [4] using GTR with a gamma distribution. Node support was calculated using the alpha likelihood ratio test. Nodes with less than 0.9 alpha likelihood ratio test support were collapsed and colored in grey. HERV-K taxa are named after their genomic location in hg19. Polymorphic HERV-Ks identified in this study are indicated in red text. The chr8:146086169 HERV-K was identified in one individual in Wildschutte *et al* [5] but not found in this analysis.
(TIF)

S5 Fig Linear discriminant analysis (LDA) based on n/T ratio of the 20 polymorphic HERV-Ks. There is improved resolution of EAS from EUR and AFR using n/T compared to reducing the data to the three states ‘provirus’, ‘solo LTR’, ‘absent’ (Fig 4) for these 20 HERV-Ks. However, there is still substantial overlap of EUR and AFR based on n/T of the 20 polymorphic HERV-K studied.
(TIF)

S6 Fig Linear discriminant analysis (LDA) using the five super populations. A) LDA plot based on the states ‘provirus’, ‘solo LTR’ and ‘absence’ of the 20 polymorphic HERV-Ks for the 5 super-populations represented in KGP. AMR are largely interspersed between AFR and EUR and SAS are found between EUR and EAS based on polymorphic status alone. B) LDA plot based on the n/T for all HERV-K proviruses for 5 super-populations. AMR and SAS overlap with EUR but are better separated from AFR based on these data.
(TIF)

S7 Fig Kernel density estimation for 12 representative polymorphic HERV-Ks. We assessed the density plots of all 96 HERV-K to determine if any peaks were specific to one of the super-populations. Shown are examples of candidate alleles specific to a population. In others several or all populations have the alleles but the prevalence is skewed. For example, the candidate allele for chr3:112743479–112752282 (the peak near n/T~0.7) appears to be more common in SAS individuals (pink trace). Similarly, EAS individuals (green trace) have a lower prevalence of the chr12:58721242–58730698 reference allele (n/T peak near 1) than do EUR (blue trace). Population-specific variation in HERV-K sequence could lead to under-estimation of proviral prevalence with mapping methods that require a coverage threshold.
(TIF)

S8 Fig Mapping four high-depth KGP individuals to the reference allele of chr1:155596457–155605636. The first row shows the positions where unique *k-mer* set T map to the reference HERV-K at chr1:155596457. The following rows show the mapping of *k-mers* recovered from four high-depth individuals: the *n/T* ratio for # 21 & 22 is equal to or close to 1; for # 20 & 23 the n/T ratio is between 0.5 and 0.7, representing a candidate allele at this locus. Note the loss of peaks at 1700bp and 3200bp in both individuals #20 and 23 and of the peak at 4700bp in #23.
(TIF)

S1 Dataset Information on HERV-K, bed files for data mining, 1000 genomes data. (XLSX)

S2 Dataset Results from analysis including matrices of n/T, presence or absence, and analysis of population prevalence and total number of HERV-K per individual. (XLSX)

S3 Dataset Analysis of co-occurrence for 3, 4, and 5 HERV-K. (XLSX)

## References

[1] Hayward A, Grabherr M, Jern P. Broad-scale phylogenomics provides insights into retrovirus-host evolution. Proc Natl Acad Sci U S A. 2013;110: 20146–51. 10.1073/pnas.1315419110 24277832 PMC3864273

[2] Feschotte C, Gilbert C. Endogenous viruses: insights into viral evolution and impact on host biology. Nat Rev Genet. Nature Publishing Group; 2012;13: 283–296. 10.1038/nrg3199 22421730

[3] Stoye JP. Studies of endogenous retroviruses reveal a continuing evolutionary saga. Nat Rev Microbiol. Nature Publishing Group; 2012;10: 395–406. 10.1038/nrmicro2783 22565131

[4] Gifford R, Tristem M. The evolution, distribution and diversity of endogenous retroviruses. Virus Genes. Springer; 2003;26: 291–315. 12876457 10.1023/a:1024455415443

[5] Weiss RA. The discovery of endogenous retroviruses. Retrovirology. 2006 10.1186/1742-4690-3-67 PMC1617120 17018135

[6] Jern P, Coffin JM. Effects of retroviruses on host genome function. Annu Rev Genet. 2008;42: 709–32. 10.1146/annurev.genet.42.110807.091501 18694346

[7] Löwer R, Löwer J, Kurth R. The viruses in all of us: characteristics and biological significance of human endogenous retrovirus sequences. Proc Natl Acad Sci. National Acad Sciences; 1996;93: 5177–5184. 8643549 10.1073/pnas.93.11.5177 PMC39218

[8] Bannert N, Kurth R. Retroelements and the human genome: New perspectives on an old relation. Proc Natl Acad Sci. 2004;101: 14572–14579. 10.1073/pnas.0404838101 15310846 PMC521986

[9] Moyes D, Griffiths DJ, Venables PJ. Insertional polymorphisms: a new lease of life for endogenous retroviruses in human disease. Trends Genet. 2007;23: 326–333. 10.1016/j.tig.2007.05.004 17524519

[10] Subramanian RP, Wildschutte JH, Russo C, Coffin JM. Identification, characterization, and comparative genomic distribution of the HERV-K (HML-2) group of human endogenous retroviruses. Retrovirology. BioMed Central Ltd; 2011;8: 90 10.1186/1742-4690-8-90 22067224 PMC3228705

[11] Wildschutte JH, Williams ZH, Montesion M, Subramanian RP, Kidd JM, Coffin JM. Discovery of unfixed endogenous retrovirus insertions in diverse human populations. Proc Natl Acad Sci. 2016; 201602336 10.1073/pnas.1602336113 PMC4843416 27001843

[12] Kurth R, Bannert N. Beneficial and detrimental effects of human endogenous retroviruses. Int J Cancer. 2010;126: 306–314. 10.1002/ijc.24902 19795446

[13] Treangen TJ, Salzberg SL. Repetitive DNA and next-generation sequencing: computational challenges and solutions. Nat Rev Genet. 2012;13: 36–46. 10.1038/nrg3117 PMC3324860 22124482

[14] Belshaw R, Watson J, Katzourakis A, Howe A, Woolven-Allen J, Burt A, et al Rate of recombinational deletion among human endogenous retroviruses. J Virol. 2007;81: 9437–42. 10.1128/JVI.02216-06 17581995 PMC1951428

[15] Medstrand P, Mager DL. Human-specific integrations of the HERV-K endogenous retrovirus family. J Virol. Am Soc Microbiol; 1998;72: 9782–9787. 10.1128/jvi.72.12.9782-9787.1998 PMC110489 9811713

[16] Hughes JF, Coffin JM. Human endogenous retrovirus K solo-LTR formation and insertional polymorphisms: implications for human and viral evolution. Proc Natl Acad Sci U S A. 2004;101: 1668–72. 10.1073/pnas.0307885100 14757818 PMC341815

[17] Belshaw R, Dawson ALA, Woolven-Allen J, Redding J, Burt A, Tristem M. Genomewide screening reveals high levels of insertional polymorphism in the human endogenous retrovirus family HERV-K (HML2): implications for present-day activity. J Virol. Am Soc Microbiol; 2005;79: 12507–12514. 10.1128/JVI.79.19.12507-12514.2005 PMC1211540 16160178

[18] Marchi E, Kanapin A, Magiorkinis G, Belshaw R. Unfixed Endogenous Retroviral Insertions in the Human Population. J Virol. 2014;88: 9529–9537. 10.1128/JVI.00919-14 24920817 PMC4136357

[19] Shin W, Lee J, Son S-Y, Ahn K, Kim H-S, Han K. Human-specific HERV-K insertion causes genomic variations in the human genome. PLoS One. Public Library of Science; 2013;8: e60605 10.1371/journal.pone.0060605 23593260 PMC3625200

[20] Gröger V, Cynis H. Human Endogenous Retroviruses and Their Putative Role in the Development of Autoimmune Disorders Such as Multiple Sclerosis. Front Microbiol. Frontiers; 2018;9: 265 10.3389/fmicb.2018.00265 29515547 PMC5826199

[21] Young GR, Stoye JP, Kassiotis G. Are human endogenous retroviruses pathogenic? An approach to testing the hypothesis. BioEssays. 2013;35: 794–803. 10.1002/bies.201300049 23864388 PMC4352332

[22] Ryan FP. Human endogenous retroviruses in health and disease: a symbiotic perspective. J R Soc Med. 2004;97: 560–5. 10.1258/jrsm.97.12.560 15574851 PMC1079666

[23] Volkman HE, Stetson DB. The enemy within: endogenous retroelements and autoimmune disease. Nat Immunol. 2014;15: 415–22. 10.1038/ni.2872 24747712 PMC4131434

[24] Magiorkinis G, Belshaw R, Katzourakis A. “There and back again”: revisiting the pathophysiological roles of human endogenous retroviruses in the post-genomic era. Philos Trans R Soc B Biol Sci. 2013;368: 20120504–20120504. 10.1098/rstb.2012.0504 PMC3758188 23938753

[25] Löwer R. The pathogenic potential of endogenous retroviruses: facts and fantasies. Trends Microbiol. Elsevier; 1999;7: 350–356. 10470042 10.1016/s0966-842x(99)01565-6

[26] Hohn O, Hanke K, Bannert N. HERV-K (HML-2), the best preserved family of HERVs: endogenization, expression, and implications in health and disease. Front Oncol. Frontiers; 2013;3: 246 10.3389/fonc.2013.00246 24066280 PMC3778440

[27] Hughes JF, Coffin JM. Human endogenous retroviral elements as indicators of ectopic recombination events in the primate genome. Genetics. 2005;171: 1183–94. 10.1534/genetics.105.043976 16157677 PMC1456821

[28] Hughes JF, Coffin JM. Evidence for genomic rearrangements mediated by human endogenous retroviruses during primate evolution. Nat Genet. Nature Publishing Group; 2001;29: 487 10.1038/ng775 11704760

[29] Romanish MT, Cohen CJ, Mager DL. Potential mechanisms of endogenous retroviral-mediated genomic instability in human cancer. Semin Cancer Biol. 2010;20: 246–253. 10.1016/j.semcancer.2010.05.005 20685251

[30] Kamp C, Hirschmann P, Voss H, Huellen K, Vogt PH. Two long homologous retroviral sequence blocks in proximal Yq11 cause AZFa microdeletions as a result of intrachromosomal recombination events. Hum Mol Genet. 2000;9: 2563–72. 11030762 10.1093/hmg/9.17.2563

[31] Kidd JM, Graves T, Newman TL, Fulton R, Hayden HS, Malig M, et al A human genome structural variation sequencing resource reveals insights into mutational mechanisms. Cell. Elsevier; 2010;143: 837–847. 10.1016/j.cell.2010.10.027 21111241 PMC3026629

[32] Cohen CJ, Lock WM, Mager DL. Endogenous retroviral LTRs as promoters for human genes: a critical assessment. Gene. Elsevier B.V.; 2009;448: 105–14. 10.1016/j.gene.2009.06.020 19577618

[33] Simmons W. The Role of Human Endogenous Retroviruses (HERV-K) in the Pathogenesis of Human Cancers. Mol Biol. 2016;05 10.4172/2168-9547.1000169

[34] Wildschutte JH, Ram D, Subramanian R, Stevens VL, Coffin JM. The distribution of insertionally polymorphic endogenous retroviruses in breast cancer patients and cancer-free controls. Retrovirology. 2014;11: 62 10.1186/s12977-014-0062-3 25112280 PMC4149278

[35] Kassiotis G, Stoye JP. Making a virtue of necessity: the pleiotropic role of human endogenous retroviruses in cancer. Philos Trans R Soc B Biol Sci. 2017;372: 20160277 10.1098/rstb.2016.0277 PMC5597744 28893944

[36] Johanning GL, Malouf GG, Zheng X, Esteva FJ, Weinstein JN, Wang-Johanning F, et al Expression of human endogenous retrovirus-K is strongly associated with the basal-like breast cancer phenotype. Sci Rep. 2017;7: 41960 10.1038/srep41960 28165048 PMC5292751

[37] Bhardwaj N, Coffin JM. Endogenous retroviruses and human cancer: Is there anything to the rumors? Cell Host Microbe. Elsevier Inc.; 2014;15: 255–259. 10.1016/j.chom.2014.02.013 24629332

[38] Hanke K, Hohn O, Bannert N. HERV-K(HML-2), a seemingly silent subtenant—but still waters run deep. Apmis. 2016;124 10.1111/apm.12475 26818263

[39] Trela M, Nelson PN, Rylance PB. The role of molecular mimicry and other factors in the association of Human Endogenous Retroviruses and autoimmunity. APMIS. 2016;124: 88–104. 10.1111/apm.12487 26818264

[40] Antony JM, Deslauriers AM, Bhat RK, Ellestad KK, Power C. Human endogenous retroviruses and multiple sclerosis: innocent bystanders or disease determinants? Biochim Biophys Acta. 2011;1812: 162–76. 10.1016/j.bbadis.2010.07.016 20696240 PMC7172332

[41] Tugnet N, Rylance P, Roden D, Trela M, Nelson P. Human endogenous retroviruses (HERVs) and autoimmune rheumatic disease: is there a link? Open Rheumatol J. 2013;7: 13 10.2174/1874312901307010013 23750183 PMC3636489

[42] Li W, Lee M-H, Henderson L, Tyagi R, Bachani M, Steiner J, et al Human endogenous retrovirus-K contributes to motor neuron disease. Sci Transl Med. 2015;7: 307ra153 10.1126/scitranslmed.aac8201 26424568 PMC6344353

[43] Douville RN, Nath A. Human Endogenous Retrovirus-K and TDP-43 Expression Bridges ALS and HIV Neuropathology. Front Microbiol. Frontiers; 2017;8: 1986 10.3389/fmicb.2017.01986 29075249 PMC5641584

[44] Trombetta B, Fantini G, D’Atanasio E, Sellitto D, Cruciani F. Evidence of extensive non-allelic gene conversion among LTR elements in the human genome. Sci Rep. 2016;6: 28710 10.1038/srep28710 27346230 PMC4921805

[45] Nexø BA, Villesen P, Nissen KK, Lindegaard HM, Rossing P, Petersen T, et al Are human endogenous retroviruses triggers of autoimmune diseases? Unveiling associations of three diseases and viral loci. Immunol Res. 2016;64: 55–63. 10.1007/s12026-015-8671-z 26091722 PMC4726719

[46] Bhardwaj N, Montesion M, Roy F, Coffin JM. Differential expression of HERV-K (HML-2) proviruses in cells and virions of the teratocarcinoma cell line Tera-1. Viruses. 2015;7: 939–68. 10.3390/v7030939 25746218 PMC4379556

[47] Fukunaga K. Introduction to statistical pattern recognition. Academic press; 2013.

[48] Ciuffi A, Ronen K, Brady T, Malani N, Wang G, Berry CC, et al Methods for integration site distribution analyses in animal cell genomes. Methods. 2009;47: 261–268. 10.1016/j.ymeth.2008.10.028 19038346 PMC4104535

[49] Witherspoon DJ, Xing J, Zhang Y, Watkins WS, Batzer MA, Jorde LB. Mobile element scanning (ME-Scan) by targeted high-throughput sequencing. BMC Genomics. 2010;11: 410 10.1186/1471-2164-11-410 20591181 PMC2996938

[50] Sudmant PH, Rausch T, Gardner EJ, Handsaker RE, Abyzov A, Huddleston J, et al An integrated map of structural variation in 2,504 human genomes. Nature. 2015;526: 75 10.1038/nature15394 26432246 PMC4617611

[51] Consortium 1000 Genomes Project, others. A global reference for human genetic variation. Nature. Nature Publishing Group; 2015;526: 68 10.1038/nature15393 26432245 PMC4750478

[52] Lin L, Chan C, West M. Discriminative variable subsets in bayesian classification with mixture models, with application in flow cytometry studies. Biostatistics. 2015;17: 40–53. 10.1093/biostatistics/kxv021 26040910 PMC4679067

[53] Escobar MD, West M. Bayesian density estimation and inference using mixtures. J Am Stat Assoc. Taylor & Francis; 1995;90: 577–588.

[54] Ishwaran H, James LF. Gibbs sampling methods for stick-breaking priors. J Am Stat Assoc. 2001;96: 161–173.

[55] Huang L, Chen H, Wang X, Chen G. A fast algorithm for mining association rules. J Comput Sci Technol. 2000;15: 619–624. 10.1007/BF02948845

[56] Benjamini Y, Hochberg Y. Controlling the false discovery rate: a practical and powerful approach to multiple testing. J R Stat Soc Ser B. 1995; 289–300.

[57] Bostock M, Ogievetsky V, Heer J. D^3^ Data-Driven Documents. IEEE Trans Vis Comput Graph. 2011;17: 2301–2309. 10.1109/TVCG.2011.185 22034350

