# Supplementary material for: A computational framework to assess genome-wide distribution of polymorphic human endogenous retrovirus-K In human populations
Source: PLoS Comput Biol. 2019 Mar 28;15(3):e1006564. doi: 10.1371/journal.pcbi.1006564 (PMC6456218; doi:10.1371/journal.pcbi.1006564)
Supplement: S1 Text — (DOCX) [file pcbi.1006564.s001.docx]

S1 Text

**Optimizing k**

A *k-mer* is a fixed length genomic sequence and the length of k is optimized for an application. We tested k= 30, 40, 50, 60, 70 to find an optimal *k* that enabled distinct clusters of both high and low depth data. It is difficult to obtain distinct clusters at low n/T values in high depth data when k=30 or 40 because of the spread of low n/T values and hard to separate the ‘present’ state in low depth data when k=70 because of a long tail at higher n/T values (S1 Fig). K= 50 or 60 separate cluster for the three states well in all data. However, k=60 takes longer processing and matching time than k=50 as *k-mer* data is larger. Thus, k was optimized to 50 because this value improved both clustering and computational efficiency.

**Dirichlet process mixture model**

The density function for the Gaussian mixture model in equation 1 is given as:

$f\left( x_{i} | \theta\right)=\sum_{j=1}^{M} \pi_{j} N(\mu_{j}, \Sigma_{j})$ , for $i=1:I,$

where in general, $x_{i}$is a d-dimensional vector observation, $\pi_{j}$ are the mixture components prior probabilities summing to 1, $M$ is the total number of Gaussian mixture components, $N(\mu_{j}, \Sigma_{j})$ is the multivariate Gaussian density for the jth component parameterized by the $d$ dimensional mean vector $\mu_{j}$ and $d\times d$ covariance matrix $\Sigma_{j}$.

The prior for $\theta$ in Eq. (1) is defined as follows:

$\pi_{1}$ = $V_{1}$, $\pi_{j}$ = ( 1 - $V_{1}$)$\cdot\cdot\cdot$(1 - $V_{j-1}$)$V_{j}$, 1 < j $\leq$ *M*,

$V_{j}$ | α ~ *B* (1, α), j= 1,…, *M*-1$; V_{M}=1$,

α ~ G(e, f),

$\mu_{j}$ | $\Sigma_{j}$ ~ $N(m, t\Sigma_{j})$,

$\Sigma_{j} \sim IW (d+2,SI_{d})$;

for pre-specified hyperparameters $(M, e, f, m, t, S)$. Here, B(.,.), G(.,.) and IW(.,.) represent the density of Beta distribution, the density of Gamma distribution and the density of Inverse-Wishart distribution, respectively. More specifically, $m$ denotes the prior mean. In Inverse Wishart distribution, $IW(d+2, SI_{d}),$ where $d$ is the dimension of the input vector (in our case $d=2$), and $SI_{d}$ is a $d\times d$ diagonal matrix where all the diagonal elements equal to *S*.

In our problem, $I= 2535$ individuals from KGP were represented by a two-dimension vector $x_{i}$= ($x_{i1}$, $x_{i2}$), where $x_{i1}$is the n/T ratio and , $x_{i2}$is the log function of depth for $i = 1,\ldots,2535$. We tuned the hyperparameters of mixture models, $(M, e, f, m, t, S),$ for better clustering in experiments. To perform clustering, the usual approach is to compute the posterior probability 𝑃($Z_{i}$=𝑗 |$x_{i}$,𝜃), and assign $x_{i}$ to the cluster $j$ with the maximum posterior probability. However, clusters can have arbitrary shapes, and the parametric distribution of each mixture component is often inadequate to capture different shapes of the clusters. Various strategies have been proposed to merge multiple mixture components so that an individual cluster can be more properly modeled [1–3]. We expect 1-5 clusters given the possible states of the HERV-K, including various types of alleles observed from density plots. We can set a larger value for $M$*,* which is the total number of mixture components needed for density estimation. Note that in the general setting,$M = 15, e = 20, f = 1, m = (0, 0)’, t = 2, d = 2, S = 5$; however, *S* can be modified to affect clustering results. It takes approximately 5 minutes to run the mixture model once for one virus on a 2.2 GHz Intel Xeon Processor with 24 CPU/server and 128 GB RAM.

**Visualization tool**

a. Query page

The left-most column of the Query page consists of a list (a dropdown menu), which is the start point for the prevalence query. This list is initialized to all polymorphic HERV-K defined in the JSON file when the interface is loaded. Users can use ‘Submit’ or ‘Join’ button to query the prevalence of the individual or set of HERV-K chosen from the dropdown menu. There is no limitation of the number of HERV-K in the JSON file. The HERV-K information in the menu is dynamically retrieved by the module.

b. Result page

The second module calculates and display the (co-)occurrence of HERV-K chosen by the user in the dropdown menus in the query page. The larger the population size is, the larger the bubble size is. Bubbles are filled in rainbow colors based on the value of insertion prevalence or co-occurrence of polymorphic HERV-K. The prevalence value in each population can also be displayed in a zoom-able way in the global map. In the right side of the interface, HERV-K prevalence or co-occurrence in 26 populations or 5 super populations are displayed. Clicking on any population displays a detailed description including population description and population

prevalence/co-occurrence will be displayed. Examples are provided in the text, Fig 3.

The code for implementing the tool is at <https://github.com/lwl1112/polymorphicHERV/tree/master/visualization>

and the tool is available with the matrix of presence, solo LTR, absence for KGB at

<http://pages.iu.edu/~wli6/visualization/>

**Determining population-specific alleles from density plots**

The n/T-depth plots (colored by super-populations, see Fig 5), for some HERV-K appear to have population specific patterns. To further investigate, we obtained a kernel density estimation for each population using the ‘density’ function in R (S7 Fig & S1 Dataset: virus).

**Code for k-mer hash table.**

The data structure for a hash of arrays for each k-mer is written in perl as follows:

%data = (

$k-mer1 => [$virus1, $virus2],

$k-mer2 => [$virus1],

…

);

**Occupancy (provirus or solo LTR) of polymorphic HERV-K.**

|  | **Occupancy (%)** | **AFR** | **AMR** | **EAS** | **EUR** | **SAS** |
| --- | --- | --- | --- | --- | --- | --- |
| **chr1:75842771** | 100.00 | 100.00 | 100.00 | 100.00 | 100.00 | 100.00 |
| **chr3:112743479** | 100.00 | 100.00 | 100.00 | 100.00 | 100.00 | 100.00 |
| **chr3:148281477** | 41.89 | 38.86 | 42.61 | 45.05 | 46.53 | 37.45 |
| **chr3:185280336** | 100.00 | 100.00 | 100.00 | 100.00 | 100.00 | 100.00 |
| **chr4:69463709** | 72.50 | 93.87 | 88.92 | 31.07 | 85.35 | 61.94 |
| **chr5:156084717** | 100.00 | 100.00 | 100.00 | 100.00 | 100.00 | 100.00 |
| **chr6:57623896** | 100.00 | 100.00 | 100.00 | 100.00 | 100.00 | 100.00 |
| **chr6:78427019** | 98.82 | 97.76 | 98.86 | 99.81 | 98.22 | 99.80 |
| **chr7:4622057** | 100.00 | 100.00 | 100.00 | 100.00 | 100.00 | 100.00 |
| **chr8:12316492** | 87.73 | 94.77 | 89.77 | 94.95 | 78.61 | 78.54 |
| **chr8:7355397** | 18.66 | 39.16 | 12.50 | 6.02 | 11.29 | 15.99 |
| **chr10:27182399** | 100.00 | 100.00 | 100.00 | 100.00 | 100.00 | 100.00 |
| **chr11:101565794** | 100.00 | 100.00 | 100.00 | 100.00 | 100.00 | 100.00 |
| **chr12:55727215** | 88.80 | 83.56 | 96.02 | 78.45 | 95.45 | 94.74 |
| **chr12:58721242** | 100.00 | 100.00 | 100.00 | 100.00 | 100.00 | 100.00 |
| **chr19:21841536** | 26.98 | 39.16 | 11.93 | 32.23 | 10.69 | 32.39 |
| **chr19:22414379** | 67.77 | 89.24 | 60.80 | 56.89 | 55.84 | 67.21 |
| **chr19:22457244** | 0.87 | 3.29 | 0.00 | 0.00 | 0.00 | 0.00 |
| **chr22:18926187** | 100.00 | 100.00 | 100.00 | 100.00 | 100.00 | 100.00 |
| **chrX:93606603** | 2.25 | 7.32 | 2.27 | 0.00 | 0.00 | 0.00 |

**References cited in the supplementary material**

1. Lin L, Chan C, West M. Discriminative variable subsets in bayesian classification with mixture models, with application in flow cytometry studies. Biostatistics. 2015;17: 40–53.

2. Li J, Ray S, Lindsay BG. A nonparametric statistical approach to clustering via mode identification. J Mach Learn Res. 2007;8: 1687–1723.

3. Finak G, Bashashati A, Brinkman R, Gottardo R. Merging Mixture Components for Cell Population Identification in Flow Cytometry. Adv Bioinformatics. Hindawi; 2009;2009: 1–12. doi:10.1155/2009/247646

4. Guindon S, Dufayard J-F, Lefort V, Anisimova M, Hordijk W, Gascuel O. New Algorithms and Methods to Estimate Maximum-Likelihood Phylogenies: Assessing the Performance of PhyML 3.0. Syst Biol. 2010;59: 307–321. doi:10.1093/sysbio/syq010

5. Wildschutte JH, Williams ZH, Montesion M, Subramanian RP, Kidd JM, Coffin JM. Discovery of unfixed endogenous retrovirus insertions in diverse human populations. Proc Natl Acad Sci. 2016; 201602336. doi:10.1073/pnas.1602336113
