# Supplementary figures and images for: A computational framework to assess genome-wide distribution of polymorphic human endogenous retrovirus-K In human populations
Source: PLoS Comput Biol. 2019 Mar 28;15(3):e1006564. doi: 10.1371/journal.pcbi.1006564 (PMC6456218; doi:10.1371/journal.pcbi.1006564)

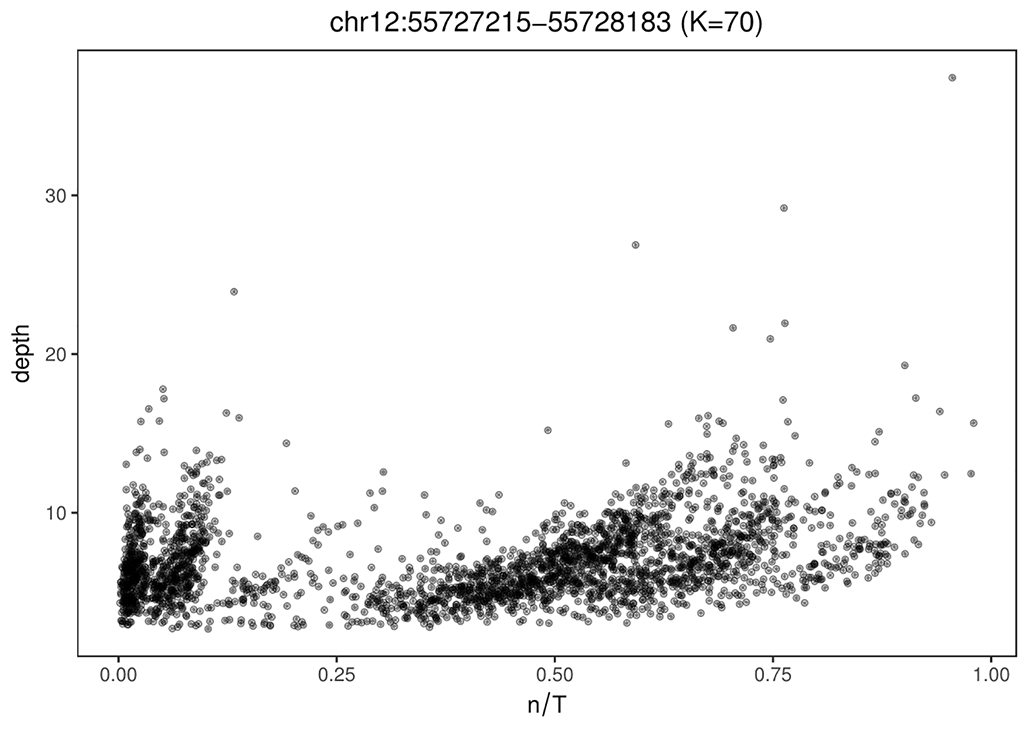

Supplement: S1 Fig — The x-axis is the n/T ratio, representing the proportion of k-mers derived from an individual’s genome data that matches the unique set T for the HERV-K at chr12:55727215–55728183. The y- axis represents sequence depth. Under these conditions, there is a tendency for clustering of some values but dispersion of points is broad and separation into biologically meaningful clusters would be difficult. For this reason, we developed the mixture model after optimizing the length k to facilitate clustering (S2 Fig and S1 Text). (TIFF) [file pcbi.1006564.s002.tiff]

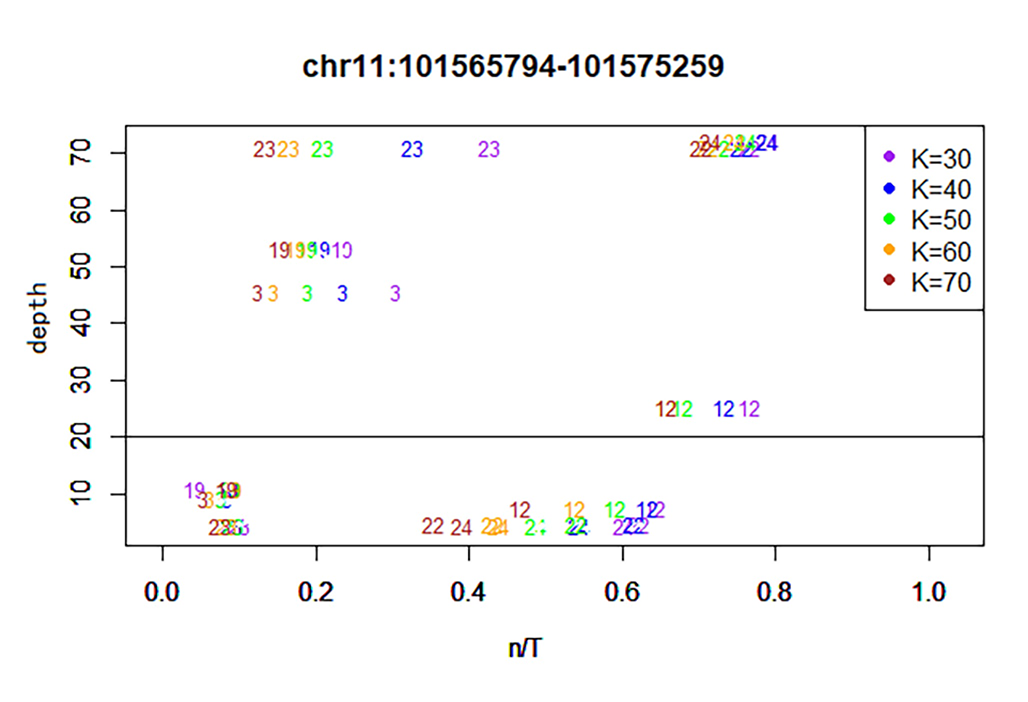

Supplement: S2 Fig — Six individuals with both high and low depth data are used to demonstrate how varying the length of k affects n/T values for absent, solo LTR and present states. High depth data is above the line (depth = 20). Different colors represent different values of k from 30–70 as shown in the legend. Each number represents a different individual (see S1 Dataset:KGP for the identify of the sample corresponding to each number). (TIF) [file pcbi.1006564.s003.tif]

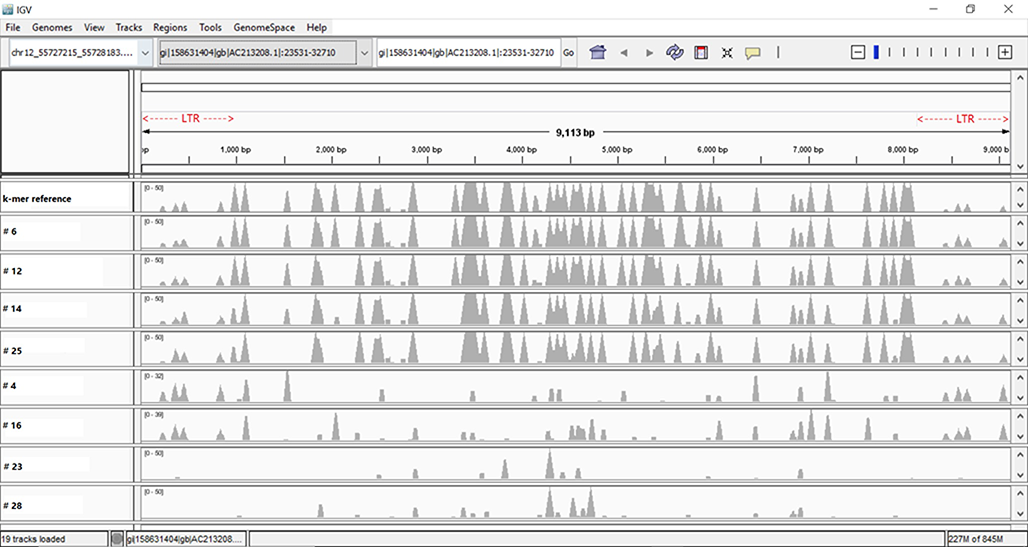

Supplement: S3 Fig — All k-mers derived from the data mining step from each individual are mapped to the reference set of unique k-mers, T, requiring 100% identity, to generate the set ‘n’ The first row shows the coverage of the set T on the HERV-K. The following plots show the mapping of the k-mer set ‘n’ from 8 individuals for the HERV-K at chr12: 55727215. # 6, 12, 14, and 25 (see S1 Dataset: KGP, column D for identification information) are labeled as ‘provirus’. Note the drop out of the peaks near 3500 and 5000bp for #14 and #25, which accounts for a decrease in n/T in these individuals. #4 and 16 have low n/T and k-mers map to the LTR region indicated above the diagram; these are labeled as ‘solo LTR’. #23, and 28 are labeled as ‘absent’. For individuals with states ‘solo LTR’ and ‘absent’, there are some peaks in the coding region. This is most likely the result of assigning unique k-mers to this HERV-K that are shared with those from a HERV-K that is absent from the reference HERV-K dataset. (TIF) [file pcbi.1006564.s004.tif]

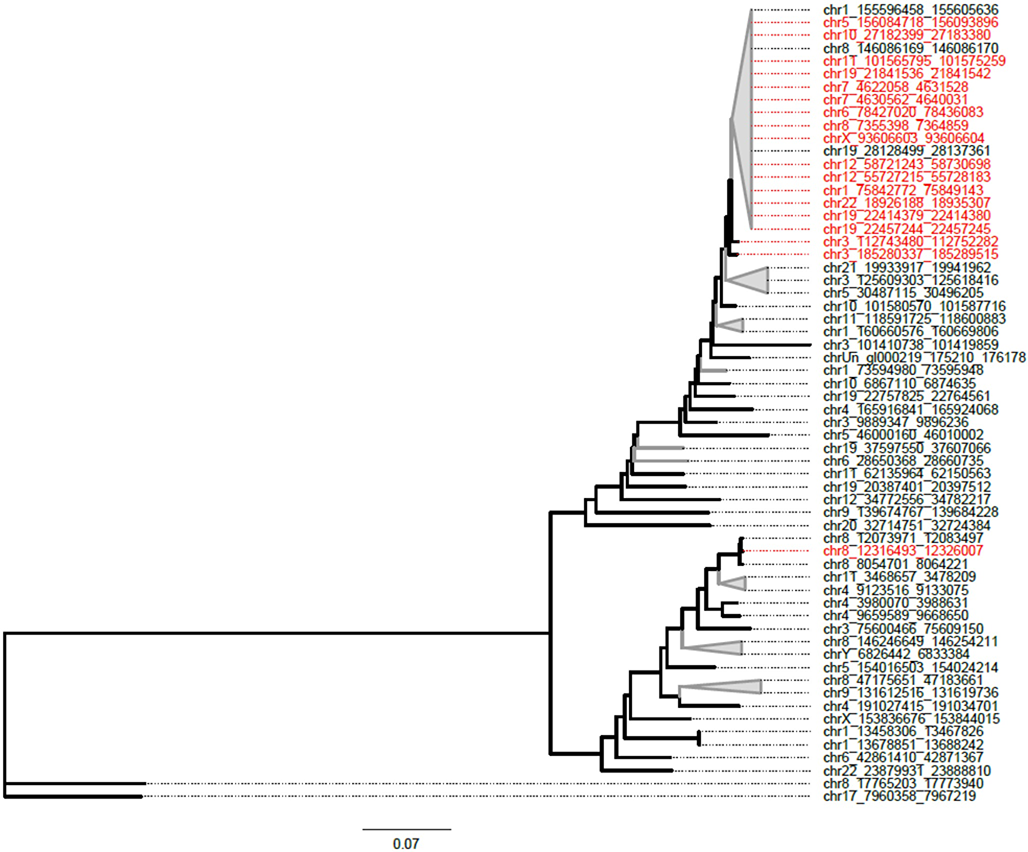

Supplement: S4 Fig — To improve the alignment, only > = 6,500 bp HERV-Ks were included except for the HERV-K at chr1:75,842,771, which has a long deletion but aligns well in other regions. Maximum likelihood tree was generated using PhyML [4] using GTR with a gamma distribution. Node support was calculated using the alpha likelihood ratio test. Nodes with less than 0.9 alpha likelihood ratio test support were collapsed and colored in grey. HERV-K taxa are named after their genomic location in hg19. Polymorphic HERV-Ks identified in this study are indicated in red text. The chr8:146086169 HERV-K was identified in one individual in Wildschutte et al [5] but not found in this analysis. (TIF) [file pcbi.1006564.s005.tif]

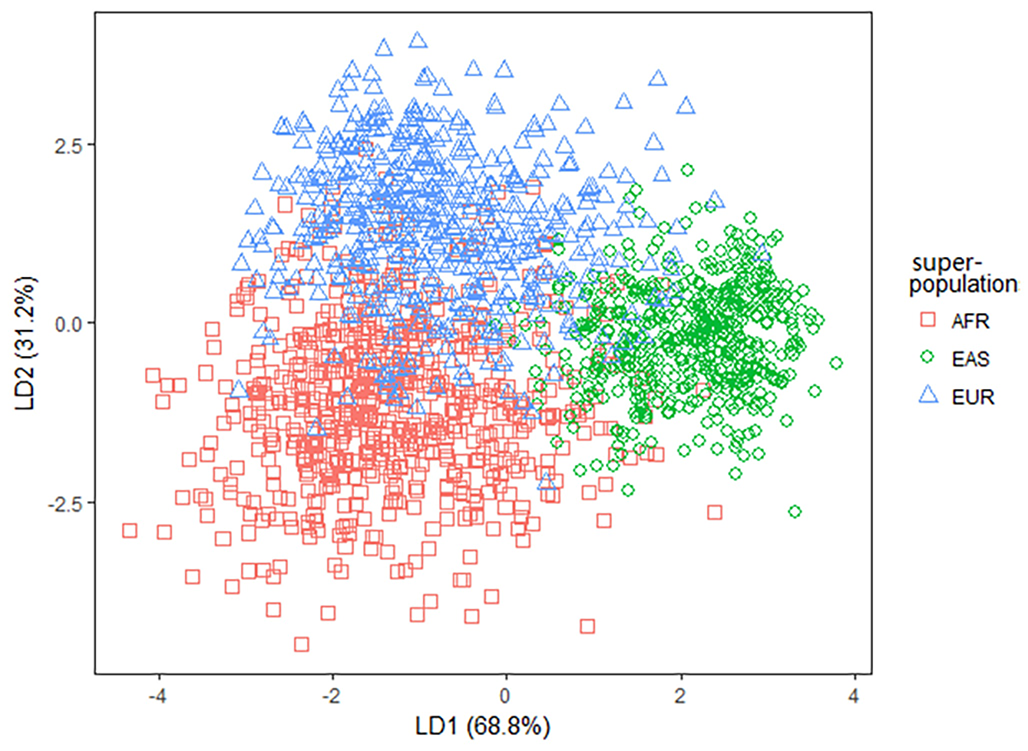

Supplement: S5 Fig — There is improved resolution of EAS from EUR and AFR using n/T compared to reducing the data to the three states ‘provirus’, ‘solo LTR’, ‘absent’ (Fig 4) for these 20 HERV-Ks. However, there is still substantial overlap of EUR and AFR based on n/T of the 20 polymorphic HERV-K studied. (TIF) [file pcbi.1006564.s006.tif]

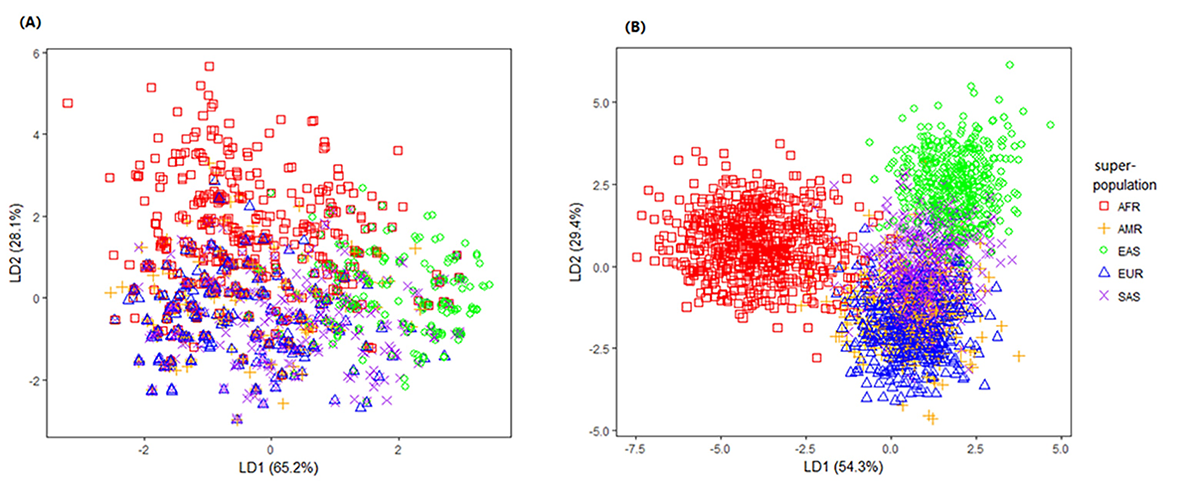

Supplement: S6 Fig — A) LDA plot based on the states ‘provirus’, ‘solo LTR’ and ‘absence’ of the 20 polymorphic HERV-Ks for the 5 super-populations represented in KGP. AMR are largely interspersed between AFR and EUR and SAS are found between EUR and EAS based on polymorphic status alone. B) LDA plot based on the n/T for all HERV-K proviruses for 5 super-populations. AMR and SAS overlap with EUR but are better separated from AFR based on these data. (TIF) [file pcbi.1006564.s007.tif]

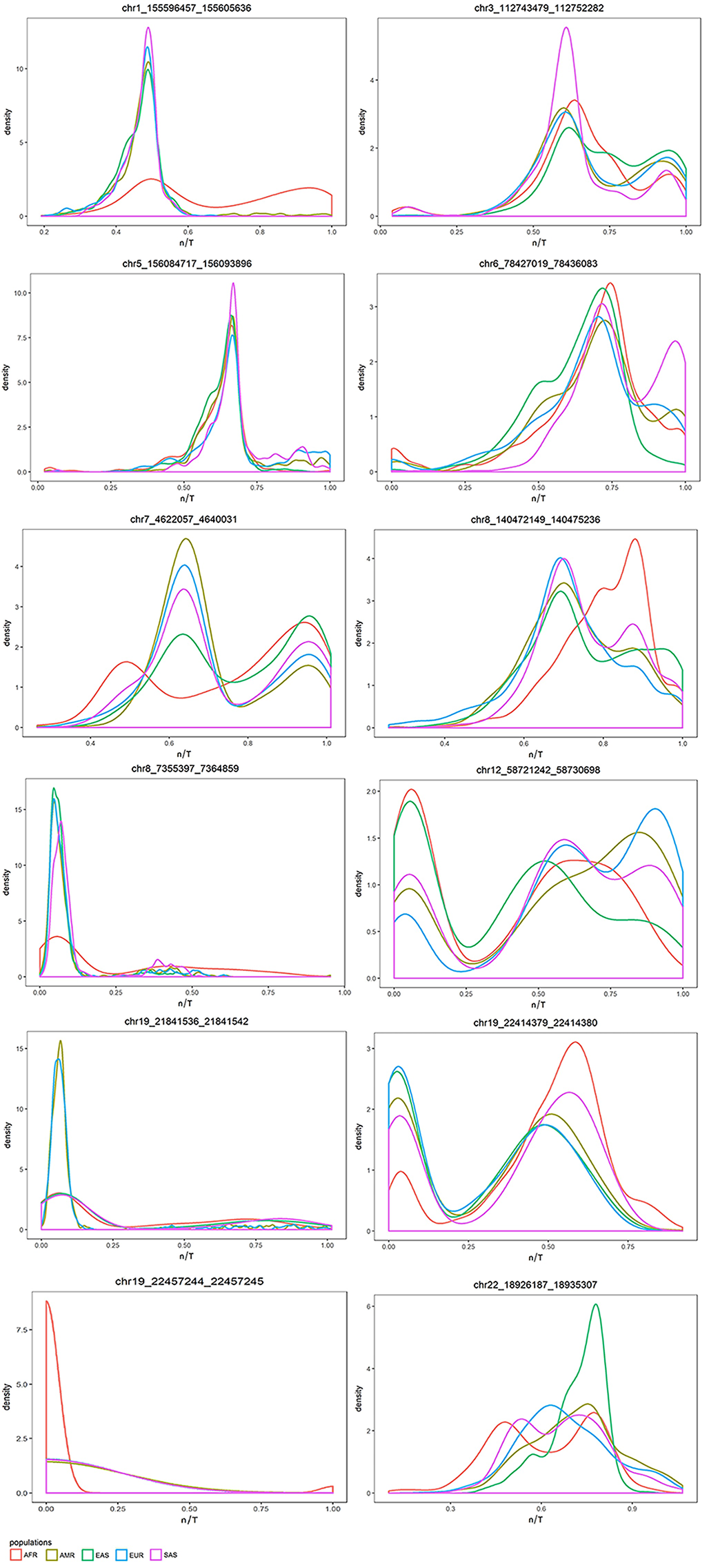

Supplement: S7 Fig — We assessed the density plots of all 96 HERV-K to determine if any peaks were specific to one of the super-populations. Shown are examples of candidate alleles specific to a population. In others several or all populations have the alleles but the prevalence is skewed. For example, the candidate allele for chr3:112743479–112752282 (the peak near n/T~0.7) appears to be more common in SAS individuals (pink trace). Similarly, EAS individuals (green trace) have a lower prevalence of the chr12:58721242–58730698 reference allele (n/T peak near 1) than do EUR (blue trace). Population-specific variation in HERV-K sequence could lead to under-estimation of proviral prevalence with mapping methods that require a coverage threshold. (TIF) [file pcbi.1006564.s008.tif]

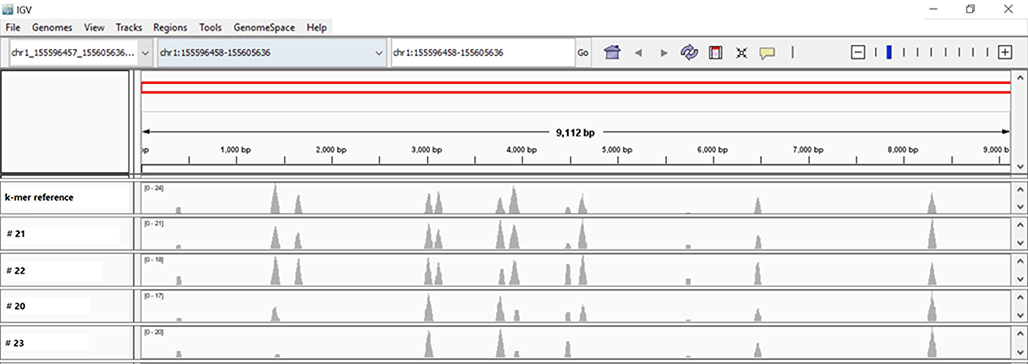

Supplement: S8 Fig — The first row shows the positions where unique k-mer set T map to the reference HERV-K at chr1:155596457. The following rows show the mapping of k-mers recovered from four high-depth individuals: the n/T ratio for # 21 & 22 is equal to or close to 1; for # 20 & 23 the n/T ratio is between 0.5 and 0.7, representing a candidate allele at this locus. Note the loss of peaks at 1700bp and 3200bp in both individuals #20 and 23 and of the peak at 4700bp in #23. (TIF) [file pcbi.1006564.s009.tif]
